# A Floating Mold Technique for the Programmed Assembly of Protocells into Protocellular Materials Capable of Non‐Equilibrium Biochemical Sensing

**DOI:** 10.1002/adma.202100340

**Published:** 2021-05-07

**Authors:** Agostino Galanti, Rafael O. Moreno‐Tortolero, Raihan Azad, Stephen Cross, Sean Davis, Pierangelo Gobbo

**Affiliations:** ^1^ School of Chemistry University of Bristol Bristol BS8 1TS UK; ^2^ Wolfson Bioimaging Facility Biomedical Sciences Building University of Bristol Bristol BS8 1TD UK

**Keywords:** bioinspiration, enzyme cascade, out‐of‐equilibrium, protocells, protocellular materials, prototissue

## Abstract

Despite important breakthroughs in bottom‐up synthetic biology, a major challenge still remains the construction of free‐standing, macroscopic, and robust materials from protocell building blocks that are stable in water and capable of emergent behaviors. Herein, a new floating mold technique for the fabrication of millimeter‐ to centimeter‐sized protocellular materials (PCMs) of any shape that overcomes most of the current challenges in prototissue engineering is reported. Significantly, this technique also allows for the generation of 2D periodic arrays of PCMs that display an emergent non‐equilibrium spatiotemporal sensing behavior. These arrays are capable of collectively translating the information provided by the external environment and are encoded in the form of propagating reaction–diffusion fronts into a readable dynamic signal output. Overall, the methodology opens up a route to the fabrication of macroscopic and robust tissue‐like materials with emergent behaviors, providing a new paradigm of bottom‐up synthetic biology and biomimetic materials science.

## Graphical abstract

Protocellular materials are constructed using a bottom‐up strategy enabled by simple floating molds. The technique allows the fabrication of free‐standing, macroscopic, and robust materials from protocell building blocks that are stable in water media, are capable of communicating both internally and with the external environment, and display rudimentary forms of emergent behaviors that are generated from the synergistic interaction of their constituent parts.

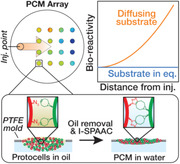

## 1 Introduction

Living tissues comprise complex 3D architectures of interconnected cell consortia that communicate and display emergent behaviors. Mimicking the structure of living tissues and understanding the physical–chemical basis of their emergent properties are two of the major goals of bottom‐up synthetic biology. Their achievement will lead to important technological advancements in tissue engineering, pharmacokinetics, personalized therapy, micro‐bioreactor technologies, and soft robotics.^[^ ^1^ ^]^

In recent years, while working toward these goals, researchers in the field of bottom‐up synthetic biology started to develop methodologies to assemble different models of synthetic protocells^[^ ^2^ ^]^ into interconnected 3D networks, termed prototissues, that communicate and display rudimental emergent behaviors.^[^ ^1f^ ^]^ For example, Bayley and co‐workers developed a 3D printing technique to pattern water‐in‐oil microdroplets connected by interface bilayers (DIBs) into synthetic tissues. They then demonstrated that DIBs are capable of membrane protein‐mediated electrical communication, macroscopic deformation, and light‐induced gene expression.^[^ ^3^ ^]^ Li et al. used magnetic fields to manipulate diamagnetic giant unilamellar lipid vesicles (GUVs) into various spatially coded configurations of a few hundred micrometers in size.^[^ ^4^ ^]^ Wang et al. showed that microarrays of hemifused GUVs could be patterned using acoustic standing waves, thus making progress toward the fabrication of prototissues with controlled geometries and lattice dimensions.^[^ ^5^ ^]^ Instead of patterning using 3D printing or by applying magnetic fields or acoustic standing waves, our group has recently developed a synthetic approach to the programmed assembly of prototissue spheroids based on the interfacial bio‐orthogonal adhesion of two populations of reactive protein–polymer protocells, termed proteinosomes.^[^ ^6^ ^]^ Proteinosomes are a well‐established protocell model and are generated using the Pickering emulsion technique. They comprise a semipermeable and elastic membrane which consists of a closely packed monolayer of conjugated bovine serum albumin/poly(*N*‐isopropylacrylamide) (BSA/PNIPAM) amphiphilic nanoparticles, and because of this they can be classified as organic colloidosomes. The BSA/PNIPAM membrane is then chemically cross‐linked with poly(ethylene glycol)‐bis(*N*‐succinimidyl succinate) (PEG‐diNHS) and the proteinosomes can be transferred into water media. Most importantly, proteinosomes can be engineered to display protocellular properties such as guest molecule encapsulation, selective permeability, gene‐directed protein synthesis, membrane‐gated internalized enzyme catalysis, predatory behaviors, and reversible contractility.^[^ ^7^ ^]^ To assemble proteinosomes into prototissue spheroids, we first synthesized a new BSA/poly(*N*‐isopropylacrylamide)‐*co*‐methacrylic acid (BSA/PNIPAM‐*co*‐MAA) nanoconjugate and functionalized it with either pendent azide or bicyclononyne (BCN) moieties. The amphiphilic bio‐orthogonally reactive protein–polymer nanoconjugates were then used to prepare two separate populations of azide‐ or BCN‐functionalized proteinosomes as water‐in‐oil (w/o) droplets using the Pickering emulsion technique. The proteinosome structures were stabilized via chemical crosslinking with PEG‐diNHS, which was pre‐dissolved in the water phase. Binary populations of the azide‐ and BCN‐functionalized proteinosomes were then spatially confined using a water‐in‐oil‐in‐water (w/o/w) Pickering emulsion procedure and structurally interlinked in situ via an interfacial strain‐promoted alkyne–azide cycloaddition (I‐SPAAC) reaction to afford prototissue spheroids 75–200 µm in diameter upon removal of the inner oil phase.^[^ ^6^ ^]^

While all these different approaches provided important breakthroughs in prototissue design and synthetic construction, they are not without their drawbacks. The w/o/w Pickering emulsion method does not provide spatial control over the protocell organization and is currently limited to the generation of prototissue spheroids with micrometer‐scale dimensions;^[^ ^6^ ^]^ acoustic patterning requires the standing waves to be constantly applied to avoid a rapid re‐dispersal of the GUVs into the bulk solution;^[^ ^5^ ^]^ the diamagnetic GUVs require an aqueous media containing high levels of MnCl_2_ and a constant magnetic field to maintain the patterns;^[^ ^4^ ^]^ and the 3D‐printing of DIBs requires the presence of an external bulk oil phase and the resulting prototissues present a very short shelf life.^[^ ^8^ ^]^ As a consequence, the possibility of using protocells as building blocks to assemble macroscopic materials that are robust, free‐standing, characterized by complex internal 3D architectures, capable of communicating both internally and with the external environment, and displaying emergent behaviors that generate from the synergistic interaction of their constituent parts still remains a considerable challenge. The development of such protocellular materials (PCMs) would open up new avenues in bottom‐up synthetic biology and bioinspired engineering and facilitate the transition of protocell research from fundamental to applied science.

As a step toward this ambitious goal, herein, we describe the first bottom‐up methodology for the fabrication of PCMs that overcomes most of the current challenges in prototissue engineering. This methodology is based on a floating poly(tetrafluoroethylene) (PTFE) mold, which can be used for the programmed assembly of millions of bio‐orthogonally reactive synthetic protocells into centimeter‐sized free‐standing tissue‐like materials of any size and shape. These PCMs are stable in water media and are capable of communicating both internally and with the external environment. Significantly, this novel floating mold technique could also be used to generate for the first time 2D periodic arrays of PCMs, which were capable of an emergent non‐equilibrium spatiotemporal sensing behavior. These arrays were capable of dynamically translating the information provided by the external environment and encoded in the form of propagating reaction–diffusion gradients into a readable signal output. In general, our work moves beyond the engineering of a strategy to generate protocell–protocell adhesions. It aims to spearhead the programmed assembly and spatial integration of different protocell phenotypes into centimeter sized free‐standing PCMs with precise architectures and geometries. Thanks to these unique characteristics, the PCMs can then combine the specialization of individual protocell types with the emergent spatiotemporal biochemical response of the ensemble, thus providing a new paradigm of bottom‐up synthetic biology and biomimetic materials science.

## 2 Results and Discussion

### 2.1 Programmed Assembly of Protocellular Materials (PCMs)

**Scheme 1:**
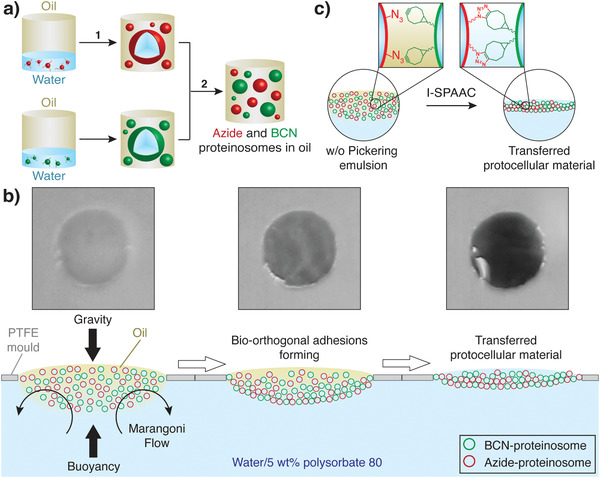
Generation of protocellular materials (PCMs). a) Scheme showing the preparation of a binary population of azide‐ (red shapes) and BCN‐functionalized (green shapes) proteinosomes in oil starting from the corresponding bio‐orthogonally reactive BSA/PNIPAM‐*co*‐MAA nanoconjugates. Step (1) involves the generation of 2 separate populations of bio‐orthogonally reactive proteinosomes in separate vials as w/o microdroplets using the Pickering emulsion technique. Step (2) involves mixing of the two populations in 1:1 ratio. b) Scheme illustrating the PCM programmed assembly process. Initially, a 1:1 binary population of azide‐ (red shapes) and BCN‐proteinosomes (green shapes) in oil prepared as described in (a) is cast inside a PTFE mold floating on a solution of polysorbate 80 in water (5 wt%). In this system the Pickering emulsion is subject to: 1) buoyancy, which keeps the emulsion inside the PTFE mold; 2) gravity, which acts to sediment the proteinosomes to the bottom of the oil droplet; and 3) Marangoni flow from the center of the PTFE mold to the sides and into the bulk solution as highlighted by the curved black arrows. With time the effect of polysorbate 80 and Marangoni flow extracts the oil from the emulsion and brings the proteinosomes in contact allowing them to react via an interfacial strain‐promoted alkyne–azide cycloaddition (I‐SPAAC) reaction and assemble the PCM. The photographs on top show the oil removal and PCM programmed assembly process on a 2 mm wide circular PTFE mold on a black background highlighting the associated opacity change from white to transparent; the appearance of black color is due to the background color. c) Scheme highlighting the I‐SPAAC reaction occurring upon oil removal.

Protein–polymer PCMs were generated from a binary population of bio‐orthogonally reactive proteinosomes in oil. First, rhodamine B isothiocyanate (RITC)‐labeled azide‐functionalized BSA/PNIPAM‐*co*‐MAA nanoconjugates (red fluorescence) and fluorescein isothiocyanate (FITC)‐labeled BCN‐functionalized BSA/PNIPAM‐*co*‐MAA nanoconjugates (green fluorescence) were synthesized using our previously established procedure.^[^ ^6^ ^]^ Subsequently, samples of RITC‐labeled azide‐ and FITC‐labeled BCN‐functionalized proteinosomes in oil (mean diameter ≈ 25 µm; mean volume ≈ 8 pL) were prepared using the Pickering emulsion technique and internally cross‐linked with PEG‐diNHS (**Scheme** **1**a; Figure S1, Section S1.2, Supporting Information).^[^ ^6^, ^7^ ^]^ Subsequently, the two populations of cross‐linked RITC‐labeled azide‐ and FITC‐labeled BCN‐functionalized proteinosomes in oil were mixed in a 1:1 ratio and drop‐cast inside a circular PTFE mold 2 mm in diameter floating on an aqueous solution of polysorbate 80 (5 wt%) to obtain a final emulsion volume of 0.64 µL mm^−2^. The binary emulsion was then allowed to transfer to the water media for ≈2 h with an associated progressive color change from white to transparent (Scheme 1b; Section S1.3, Supporting Information).

**Figure 1:**
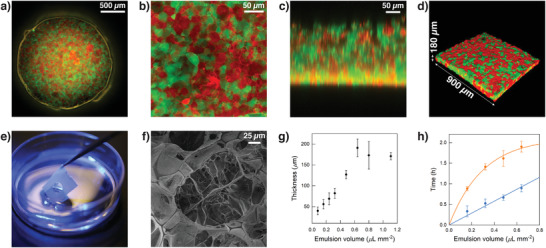
PCM characterization. a) Tiled epifluorescence microscopy image of a circular PCM 2 mm in diameter attached to a PTFE mold and immersed in an aqueous solution of polysorbate 80 (5 wt%). The PCM comprises an interlinked 1:1 binary population of FITC‐labeled (green fluorescence) BCN‐functionalized and RITC‐labeled (red fluorescence) azide‐functionalized proteinosomes internally cross‐linked with PEG‐diNHS. The PCM was prepared by adding an emulsion volume of 0.64 µL mm^−2^. b) *XY* confocal fluorescence microscopy image showing a zoomed in area of the PCM in (a). c) *XZ* confocal fluorescence microscopy scan showing a zoomed in vertical section of the PCM in (a). d) 3D confocal image of the PCM in (a). The image shows that the PCM has a homogeneous thickness of ≈180 µm. Images in (b), (c), and (d) highlight the formation of a spatially interlinked network of closely packed bio‐orthogonally ligated proteinosomes. e) Photograph demonstrating the robustness and ease of lifting the PTFE mold with attached PCM from the aqueous solution of polysorbate 80 (5 wt%). In this image a water meniscus crossing the circular mold can be noted, highlighting the presence of the PCM inside. f) Scanning electron microscopy (SEM) image of a freeze‐dried free‐standing PCM showing the details of the spatially interlinked network of closely packed bio‐orthogonally ligated proteinosomes. g) Graph showing changes in the PCM thickness as a function of the emulsion volume used to assemble them. Data obtained from the analysis of Figure S3, Supporting Information. Error bars: standard deviation. h) Graph showing onset of transfer time (blue plot) and final transfer time (orange plot) as a function of the emulsion volume used to assemble the PCMs, ranging between 0.16 and 0.64 µL mm^−2^. Data obtained from the analysis of Figure S4, Supporting Information. Error bars: standard deviation.

Time‐dependent fluorescence microscopy imaging showed that PCMs formed via a progressive oil removal process with concomitant bio‐orthogonal ligation of the binary proteinosome population (Scheme 1c; Video S1, Supporting Information). This resulted in membrane‐bounded PCMs with a spatially integrated tissue‐like structure that remained attached to the PTFE mold, as shown by fluorescence microscopy imaging (**Figure** **1**a). Confocal fluorescence microscopy showed that the PCMs prepared with an emulsion volume of 0.64 µL mm^−2^ had a homogeneous thickness of ≈180 µm (Figure 1b–d). Significantly, the connection between the PCM and the PTFE mold was strong enough to allow for the mold to be lifted from the aqueous solution and for the sample to be handled in air (Figure 1e). The PCM could also be easily detached from the mold, resulting in free‐standing PCMs in water media that could be manipulated with tweezers. This allowed for the preparation of samples for scanning electron microscopy (SEM) imaging, which showed tightly interconnected protein–polymer cell‐like structures resembling plant tissue or squamous epithelium tissue, highlighting the free‐standing nature of the material Figure 1f; Figure S2, Supporting Information).

The thickness of the PCMs could be controlled by varying the volume of the 1:1 binary emulsion. In a series of systematic experiments, we generated circular PCMs 2 mm in diameter by progressively increasing the emulsion volume from 0.08 (minimum volume we could inject) to 1.10 µL mm^−2^ (maximum volume that could reproducibly be contained in the mold) and used confocal fluorescence microscopy to characterize the thickness of the different PCMs. The PCM thickness could be varied from a minimum of 40 ± 9 µm (i.e., a mono/bilayer of proteinosomes) to a maximum of 190 ± 20 µm (Figure 1g; Figure S3, Supporting Information). The thickness was found to increase linearly with the emulsion volume between 0.08 and 0.64 µL mm^−2^ and reached a plateau at higher volumes due to the overflow of the emulsion from the bottom of the mold. Moreover, PCMs rich in voids formed using emulsion volumes < 0.32 µL mm^−2^. 0.32 µL mm^−2^ was the minimum emulsion volume needed to obtain a continuous, non‐defective PCM 80 ± 10 µm thick (Figure S3d, Supporting Information).

Due to the color change from white to transparent, the oil removal and PCM programmed assembly process could be monitored using a digital camera to obtain PCM transfer curves as a function of the emulsion volume used to assemble the PCMs, ranging between 0.16 and 0.64 µL mm^−2^ (Section S1.4, Figure S4, Video S2, Supporting Information). The PCM transfer curves displayed a sigmoidal shape, and the onset time of the curve was found to increase linearly with the emulsion volume used (1h, blue plot). In contrast, the final PCM transfer time, defined as the intersection of the slope of the sigmoidal curve and the plateau region, displayed a logarithmic growth (Figure 1h, orange plot). These observations seem to indicate that the onset of the oil removal process depends linearly on the volume of 2‐ethyl‐1‐hexanol present in the sample, but the rate of diffusion of the oil into the bulk solution tends to reach a threshold value at high emulsion densities. Moreover, the importance of polysorbate 80 in the oil removal and PCM programmed assembly process was also highlighted by control experiments carried out in the absence of the surfactant. Under this condition a strong osmotic pressure across the PCM caused the growth of large water bubbles on top of the PCM with concomitant PCM deformation and rupture when the bubbles reached a critical size (Video S3, Supporting Information). This still allowed for a slow transfer of the prototissue into water (≈8 h, Figure S5, Supporting Information), but the resultant PCM proved more fragile and inhomogeneous. In comparison, in the presence of polysorbate 80, the transfer of the PCM into water occurred in ≈1 h (Figure S5, Supporting Information), highlighting the key role of the surfactant in the PCM formation.

Significantly, control experiments carried out using non‐bio‐orthogonally reactive proteinosomes highlighted the critical role of bio‐orthogonal chemistry in the PCM generation. Video S4, Supporting Information, compares the oil removal and PCM programmed assembly process of two experiments performed in parallel using normal (non‐bio‐orthogonally reactive) proteinosomes (left) and bio‐orthogonally reactive proteinosome (right). In the absence of bio‐orthogonal ligation the Marangoni flow pushed proteinosomes to the edge of the mold and dragged them into the bulk solution (Scheme 1), resulting at best in the formation of a thin and defective PCM (Video S4, Supporting Information, left). In contrast, in the presence of bio‐orthogonal ligation, as soon as the oil was removed and azide‐ and BCN‐functionalized proteinosome entered in contact, they promptly reacted via the I‐SPAAC reaction and formed a spatially integrated tissue‐like structure (Video S4, Supporting Information, right). Moreover, attempts to generate PCMs in the absence of the PTFE mold were unsuccessful as once the emulsion touched the aqueous solution it readily spread into individual proteinosomes under the action of the Marangoni flow. The individual proteinosomes then transferred to the aqueous phase without assembling into PCMs.

Taken together, these observations seem to indicate that the PCM assembly process involves a synergistic effect of the mold (holding in place the bio‐orthogonally reactive proteinosomes in oil), the surfactant‐mediated oil removal, the Marangoni flow, and the bio‐orthogonal ligation. Most importantly, our new floating mold technique allows us to assemble proteinosome building blocks together into macroscopic and free‐standing PCMs with controllable thickness that are mechanically robust and stable in water media for months.

### 2.2 Generation of PCMs with Complex 3D Architectures

Having established that the floating mold method can be successfully used to generate macroscopic and free‐standing tissue‐like materials from a binary community of bio‐orthogonally reactive proteinosomes, we next explored its versatility for the generation of PCMs with complex 3D architectures.

**Figure 2:**
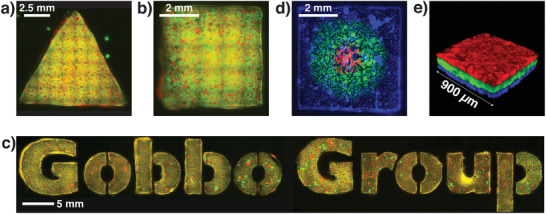
Generation of PCMs with complex 3D architectures. a) Tiled epifluorescence microscopy image of a PCM in the shape of an equilateral triangle with 1.0 cm sides. The PCMs comprised an interlinked 1:1 binary population of RITC‐labeled (red fluorescence) azide‐ and FITC‐labeled (green fluorescence) BCN‐functionalized proteinosomes internally cross‐linked with PEG‐diNHS. b) Tiled epifluorescence microscopy image of a PCM in the shape of a square with 5 mm sides with the same composition as the PCM in (a). c) Tiled epifluorescence microscopy images showing PCMs with the same composition as the PCM in (a) and composing the “Gobbo Group” logo. d) Tiled epifluorescence microscopy image of a patterned squared PCM with sides of 5 mm comprising interlinked 1:1 binary populations of non‐tagged azide‐ and differently tagged BCN‐functionalized proteinosomes internally cross‐linked with PEG‐diNHS. Blue fluorescence: Dylight405; green fluorescence: FITC; and red fluorescence: RITC. The patterns were manually generated using a mechanical pipette. e) 3D confocal fluorescence image of a 3‐tiered stratified PCM ≈270 µm thick. All layers are composed of an interlinked 1:1 binary population of BCN‐ and azide‐functionalized proteinosomes internally cross‐linked with PEG‐diNHS. Blue fluorescence: Dylight405; green fluorescence: FITC; and red fluorescence: RITC. The 3 PCM layers were perfectly attached to each other and no delamination was observed; see also Figure S11, Supporting Information.

First, we explored the possibility of generating PCMs of different shapes and sizes. As a step toward this goal, we built a PTFE mold in the shape of an equilateral triangle with 1.0 cm sides and a PTFE mold in the shape of a square with 0.5 cm sides and used them to generate PCMs at a 0.64 µL mm^−2^ emulsion volume. Epifluorescence microscopy images showed the successful programmed assembly of defect‐free PCMs in the shape of a triangle (**Figure** **2**a) and of a square (Figure 2b) of the desired dimensions. Neither of the PCMs presented cracks or defects, both fully transferred to the water phase and remained attached to the PTFE mold. Despite the large area (43.3 and 25.0 mm^2^, respectively), both PCMs could be easily lifted from the surfactant solution using the mold and transferred to a different solution without breaking. Significantly, since the PCM shape is defined by the PTFE mold and this can be laser‐cut using a high‐precision CNC machine, there is virtually no limitation to the complexity of PCM shapes that can be fabricated with this method. To demonstrate this, we constructed different PTFE molds with our research group's logo (size: 6.5 × 2.2 cm) using 3 different text fonts and used them to assemble PCMs with complex shapes. Epifluorescence microscopy imaging showed the successful programmed assembly of millions of RITC‐ and FITC‐labeled bio‐orthogonally reactive proteinosomes into the Gobbo Group's logo (Figure 2c; Figures S6–S8, Supporting Information).

Next, we explored the possibility of generating patterns of different proteinosome populations within the same PCM. To achieve this, we synthesized different populations of BCN‐ and azide‐functionalized proteinosomes in oil: 1) non‐labeled azide‐functionalized proteinosomes, 2) Dylight405‐labeled BCN‐functionalized proteinosomes, 3) FITC‐labeled BCN‐functionalized proteinosomes, and 4) RITC‐labeled BCN‐functionalized proteinosomes. These populations were then used to generate 3 binary populations of proteinosomes by mixing the non‐labeled azide‐functionalized proteinosomes in a 1:1 ratio with the Dylight405‐, FITC‐, or RITC‐labeled BCN‐functionalized proteinosomes. Patterned PCMs with circular and concentric green and red fluorescent proteinosome populations on a background of blue fluorescent proteinosomes were then generated by manual patterning of the 3 differently labeled binary populations of proteinosomes in oil inside a 0.5 cm wide square PTFE mold using a mechanical pipette. The patterned emulsions were then allowed to transfer to the aqueous phase and assemble into the patterned PCM (Section S1.5, Supporting Information). Epifluorescence microscopy imaging showed successful formation of the desired PCM with circular concentric patterns of proteinosome consortia Figure 2d; Figure S9, Supporting Information). Most importantly, no noticeable differences were observed in the pattern when the PCM was flipped upside‐down and imaged (Figure S9, Supporting Information). This indicated that the technique produced patterns that were homogenous through the PCM thickness, that is, the pattern remained in the *xy* plane and no stacking of the proteinosome populations was observed. Patterned PCMs with 2 × 2 and 3 × 3 arrays of red fluorescent proteinosome populations on a background of green fluorescent proteinosomes were also generated. The arrays were achieved by manual patterning of a binary population of RITC‐labeled azide‐ and BCN‐functionalized proteinosomes in oil on a background emulsion comprising a binary population of FITC‐labeled azide‐ and BCN‐functionalized proteinosomes in oil. The patterned emulsions were then allowed to transfer to the aqueous phase and assemble into the patterned PCM (Section S1.5, Supporting Information). Epifluorescence microscopy imaging showed successful formation of the desired PCMs with 2 × 2 and 3 × 3 arrays of proteinosome consortia (Figure S10, Supporting Information). Stratified PCMs could then be generated using a layer‐by‐layer technique. First, we cast a binary population of Dylight405‐labeled bio‐orthogonally reactive proteinosomes in oil and allowed them to transfer into water and assemble into a first PCM layer ≈90 µm thick. The further 2 proteinosome layers could then be cast on top this first layer simply by repeating the same protocol using different fluorescently labeled binary populations of bio‐orthogonally reactive proteinosomes in oil. Upon transfer, each proteinosome layer adhered to the layer underneath via an inter‐layer I‐SPAAC reaction, resulting, overall, in a stratified prototissue *≈*270 µm thick. Confocal fluorescence microscopy imaging showed that all layers were homogenous in thickness and no layer delamination was observed (Figure 2e; Figure S11, Supporting Information). Furthermore, we did not observe any difference in the oil removal process when preparing single‐layered or stratified PCMs. We ascribed this to the high permeability of the proteinosome membrane to small molecules, which made the oil extraction process very effective even in the presence of transferred PCM layers.

Overall, these results demonstrate the high versatility of our floating mold technique. Our novel approach is extremely promising and pioneers a route to the design and synthetic construction of PCMs of large size and of any shape that are stable in water media, and comprise patterns and layers of different protocell consortia.

### 2.3 Non‐Equilibrium Biochemical Sensing in PCMs

**Figure 3:**
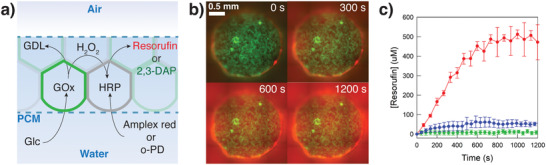
Communication properties of PCMs. a) Scheme representing the GOx/HRP enzyme cascade reaction in a PCM (enclosed by the 2 blue dashed lines) consisting of GOx‐containing BCN‐functionalized proteinosomes (green shapes) and HRP‐containing azide‐functionalized proteinosomes (grey shapes). The substrates glucose (Glc) and Amplex red or *o*‐phenylenediamine (*o*‐PD) freely diffuses through the PCM. The GOx‐containing protocells oxidize Glc to d‐glucono‐1,5‐lactone (GDL) and H_2_O_2_. This initiates radial diffusion of H_2_O_2_ from the GOx‐containing protocells, which is then used by HRP‐containing protocells to oxidize the non‐fluorescent molecules, Amplex red or *o*‐PD to red fluorescent resorufin or green fluorescent 2,3‐diaminophenazine (2,3‐DAP), respectively. H_2_O_2_ can therefore be considered as a signaling molecule between the two interlinked protocell communities. b) Time‐dependent epifluorescence microscopy images of a circular PCM 2 mm in diameter prepared as described in (a) and in the presence of glucose and Amplex Red (20 and 0.5 × 10^−3^ m in PBS 10 × 10^−3^ m, pH 6.8, respectively) at 25 °C. Green fluorescence, GOx‐containing FITC‐labeled BCN‐functionalized proteinosomes; red fluorescence, resorufin production. c) Graph showing the time‐dependent generation of resorufin from a circular PCM 2 mm in diameter and structured as described in (a) (red curve), in the absence of GOx (control experiment, blue curve), and in the absence of HRP (control experiment, green curve). Experiment repeated in triplicate; error bars: standard deviation.

Inspired by the above observations, we extended our methodology to construct the first PCMs capable of supporting a collective and coordinated spatiotemporal biochemical response via internally derived molecule‐based signaling. As a first step toward this goal, we investigated whether the PCMs were capable of sensing the external environment and triggering a coordinated internalized cascade of chemical signals via enzyme catalysis. To achieve this, we prepared a circular PCM 2 mm in diameter from a binary population of FITC‐labeled BCN‐functionalized and non‐labeled azide‐functionalized proteinosomes that were preloaded with glucose oxidase (GOx) and horseradish peroxidase (HRP), respectively (Sections S1.7 and S1.8, Supporting Information). Once transferred to the water media, the PCM was moved to a Petri dish containing an aqueous solution of glucose (Glc, 20 × 10^−3^ m) and Amplex Red (0.5 × 10^−3^ m). This initiated a spatially coupled GOx/HRP enzyme cascade reaction under diffusional equilibrium conditions between the 2 bio‐orthogonally interlinked protocell populations. The GOx containing protocells converted Glc to d‐glucono‐1,5‐lactone (GDL) and internally produced the signaling molecule H_2_O_2_, which was used to communicate to the HRP‐containing protocells to oxidize Amplex Red to resorufin and produce a fluorescent signal (**Figure** **3**a). Epifluorescence microscopy was used to determine the location of the GOx‐containing FITC‐labeled BCN‐functionalized proteinosomes within the PCM and monitor the onset and in situ development of red fluorescence due to the endogenous production of resorufin (Figure 3b; Video S5, Supporting Information). Typically, the onset of red fluorescence in the HRP‐containing protocells occurred within the first minute, followed by diffusion into the neighboring GOx‐containing protocells and external environment. By contrast, control experiments involving PCMs lacking either GOx or HRP showed no fluorescence increase due to the inability of these PCMs to sense glucose in the surrounding environment or synthesize resorufin, respectively (Figure 3c).

**Figure 4:**
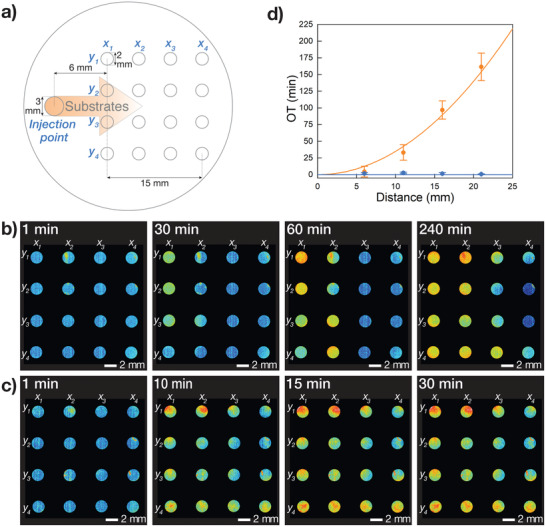
Non‐equilibrium biochemical sensing in 4 × 4 arrays of enzymatically active PCMs. a) Scheme showing the circular PTFE mold used for the non‐equilibrium biochemical sensing experiments. The scheme highlights the injection point, the *x*_1–4_ *y*_1–4_ wells used for the assembly of the 4 × 4 array of enzymatically active PCMs, and the direction of the unidirectional diffusion front of chemical substrates (orange arrow). b) Sequence of false color epifluorescence microscopy images showing spatiotemporal response of a 4 × 4 PCM array of enzymatically active PCMs, which was exposed to a co‐diffusing mixture of Glc and *o*‐PD substrates. The images show a consecutive fluorescence turn‐on of columns *x*_1–4_ associated with the in situ production of 2,3‐DAP. See Section S1.9, Supporting Information, for experimental details. c) Sequence of false color time‐dependent epifluorescence microscopy images showing the control experiment performed on a 4 × 4 PCM array of enzymatically active PCMs under diffusional equilibrium conditions. The images show a simultaneous fluorescence turn‐on of all PCMs in the array. See Section S1.9, Supporting Information, for experimental details. d) Plot showing the trend of average onset times (OTs) of 2,3‐DAP fluorescence for each *x*_1–4_ column of the 4 × 4 PCM array as a function of the distance from the injection point obtained for the experiments in (b) (orange plot) and (c) (blue plot). The orange plot highlights a quadratic relationship between the average OTs and the distance from the injection point, which is typical for diffusing chemical species. In contrast, the blue plot shows that the average OTs is independent of the spatial position of the PCMs.

Given the PCM's ability to sense and respond to chemical changes in the environment, we next displayed the potential of our floating mold technique by assembling, for the first time, 2D arrays of spatially encoded PCMs that could detect and visualize advancing concentration fronts of chemical gradients under non‐equilibrium conditions. To achieve this, we first constructed a new PTFE mold featuring a 4 × 4 array of circular wells for PCM production and an injection point placed on the left‐hand side of the array (**Figure** **4**a). This PTFE mold was utilized to assemble a 4 × 4 array of enzymatically active circular PCMs 2 mm in diameter (Section S1.9, Supporting Information). Each PCM comprised an interlinked 1:1 binary population of non‐labeled GOx‐containing BCN‐functionalized proteinosomes and HRP‐containing azide‐functionalized proteinosomes. Subsequently, the PTFE mold loaded with the array of PCMs was allowed to float on 0.980 mL of a phosphate buffer solution (PBS, 10 × 10^−3^ m, pH 6.8). The PCM array was then exposed to a left‐to‐right unidirectional reaction–diffusion gradient by injecting 20 µL of an aqueous solution of glucose (Glc, 100 × 10^−3^ m) and *o*‐phenylenediamine (*o*‐PD, 50 × 10^−3^ m) through the injection point on the left‐hand side of the PCM array. This induced spatiotemporal oxidase/peroxidase responses to the co‐diffusion of Glc and *o*‐PD substrates across the periodically ordered enzymatically active PCM array. The array's spatiotemporal responses could be followed using time‐dependent fluorescence microscopy by monitoring the development of green fluorescence associated with the HRP‐mediated oxidation of *o*‐PD to 2,3‐diaminophenazine (2,3‐DAP) in the azide‐functionalized proteinosomes. In general, a wave of 2,3‐DAP production unidirectionally moved across the PCM array from left to right. We associated this with the progressive co‐diffusion of Glc and *o*‐PD substrates (Figure 4b, Video S6, Supporting Information). Time‐dependent mean fluorescence intensity analysis showed that the onset time of fluorescence (OT) increased quadratically across the rows of the array oriented parallel to the substrate diffusion front (Figure 4d), whereas it showed minimal difference across the columns oriented perpendicularly to the substrate diffusion front (Table S2, Figure S12, Supporting Information). A similar trend was found for the initial rates of 2,3‐DAP production, which gradually diminished across the rows placed parallel to the direction of the substrate diffusion front and showed comparable rates across the columns placed perpendicular to the substrate diffusion front. The fluorescence associated with each individual PCM was found to increase to a steady state and then slowly decrease in intensity. We attributed this to the consumption of the substrates and to the diffusion of the 2,3‐DAP into the bulk solution. Taken together, these observations indicate that the substrates were progressively depleted as the reaction–diffusion front advanced through the PCM array from left to right. We also noticed that PCMs *x*_3_ *y*_1_, *x*_4_ *y*_1_, and *x*_3_ *y*_4_, *x*_4_ *y*_4_ always developed a higher mean fluorescence intensity compared to PCMs *x*_3_ *y*_2_, *x*_4_ *y*_2_, and *x*_3_ *y*_3_, *x*_4_ *y*_3_ (Video S6, Figure S12 blue and green plots, Supporting Information). We attributed this to the PCMs in rows *y*_1_ and *y*_4_ being exposed to additional Glc diffusing along the top and the bottom of the field of view. By contrast, a control experiment carried out under diffusional equilibrium conditions where the enzymatically active 4 × 4 PCM array was placed on a PBS solution (10 × 10^−3^ m, pH 6.8) preloaded with both Glc and *o*‐PD (final concentrations 1.0 and 0.5 × 10^−3^ m, respectively) showed a nearly immediate homogeneous fluorescence turn‐on through the entire PCM array (Figure 4c; Video S7, Figure S13, Supporting Information). The onset time of fluorescence of all 16 PCMs took place during the first 2–3 min of the experiment, and, as expected, it was independent of the spatial position of the PCMs (Figure 4d; Table S3, Supporting Information). The time‐dependent mean fluorescence intensity curves reached a maximum after ≈60 min, which was followed by a progressive decrease of the signal due to the depletion of the substrates and diffusion of the 2,3‐DAP product into the bulk solution (Figure S13, Supporting Information). These observations are coherent with our initial hypothesis that 2D arrays of PCMs can be biochemically programmed to collectively detect and visualize advancing concentration gradients of substrates of interest under non‐equilibrium conditions.

In order to improve our understanding of the transient spatiotemporal response of our enzymatically active 4 × 4 array of PCM, we performed additional experiments by single‐component diffusion of either *o*‐PD or Glc into a solution preloaded with the second substrate, that is, Glc or *o*‐PD, respectively. As was previously the case, in both experiments time‐dependent mean fluorescence intensity analysis in general showed a propagating fluorescence wave that moved through the PCM array from left to right (Videos S8 and S9, Supporting Information). This was associated with the progressive diffusion of the *o*‐PD or Glc and with the HRP‐mediated production of 2,3‐DAP. However, by comparing these two different experiments, we also noticed some important differences in the spatiotemporal response of the 4 × 4 PCM array. When we preloaded Glc and diffused *o*‐PD through the PCM array we observed a slow sequential fluorescence turn‐on of columns *x*_1_ and *x*_2_, whereas columns *x*_3_ and *x*_4_ did not turn on in the timeframe of the experiment (Figure S14, Supporting Information). Moreover, columns *x*_1_ and *x*_2_ continued to produce 2,3‐DAP during the timeframe of the experiment and they did not turn off as in the previous experiment where we diffused both Glc and *o*‐PD. These observations are consistent with the rate limiting step of the overall process being the production of H_2_O_2_, rather than the diffusion of *o*‐PD through the array. This was attributed to a low concentration of preloaded Glc in the system and to the slower catalytic reactivity of the GOx‐containing protocells compared to those containing HRP. The latter was ascribed to a lower molar loading of GOx in the protocells compared to HRP. However, when we diffused Glc in a bulk solution pre‐loaded in *o*‐PD we observed a fast and sequential fluorescence turn‐on of the entire array from columns *x*_1_ to *x*_4_, followed by a similarly fast and sequential decrease of the fluorescence signal in each PCM (Figure S15). We attributed this behavior to a fast local production of H_2_O_2_ when the concentrated diffusion front of Glc reached the PCM columns, followed by a fast local depletion of the *o*‐PD substrate due to the high activity of the HRP‐containing protocells that composed the PCMs. Since in this instance the rate limiting step of the overall process was the diffusion of Glc, this allowed for the estimation of a rate of diffusion for Glc of 9.1 (±0.2) × 10^−8^ m^2^ s^−1^ under these experimental conditions (Figure S16, Supporting Information). In this experiment we also observed that PCMs *x*_3_ *y*_1_, *x*_4_ *y*_1_, and *x*_3_ *y*_4_, *x*_4_ *y*_4_ had a higher mean fluorescence intensity compared to PCMs *x*_3_ *y*_2_, *x*_4_ *y*_2_ and *x*_3_ *y*_3_, *x*_4_ *y*_3_ (Video S9, Supporting Information). This was consistent with what was observed in the previous experiment where we co‐diffused Glc and *o*‐PD and was due to the PCMs in rows *y*_1_ and *y*_4_ being exposed to additional Glc diffusing along the top and the bottom of the field of view.

Overall, these experiments demonstrate that PCM arrays provide a novel chemically programmable framework in which to systematically study information encoded in propagating reaction–diffusion gradients of chemicals, such as direction of the diffusing front, spatiotemporal changes in chemical concentrations, estimation of the diffusion rates of chemical species, and identification of rate‐limiting steps of the PCM bioreactivity. These results therefore provide a first important example of spatially organized prototissues that can sense the external environment, trigger an endogenous coordinated response, and operate under non‐equilibrium conditions, providing a new paradigm of prototissue engineering.

## 3 Conclusions

Working toward fully autonomous synthetic tissues, we used bio‐orthogonal chemistry for the programmed assembly of synthetic protocells into centimeter‐sized tissue‐like materials that are stable in water media, can communicate internally and with the external environment, and are capable of emergent non‐equilibrium biochemical sensing. This was achieved by packing millions of bio‐orthogonally reactive proteinosomes in oil at the water‐air interface inside a PTFE mold floating on a 5 wt% solution of polysorbate 80. Robust PCMs were generated from the synergistic effect of surfactant‐mediated oil removal, Marangoni flow, and interfacial bio‐orthogonal ligation of the protocell building blocks.

We then showed that PCMs with complex 3D architectures could be easily constructed using this novel floating mold technique and we successfully generated patterns of different protocell phenotypes and stratified PCMs. It should now be possible to advance this methodology to generate even more complex 3D architectures where protocell populations with different specialized functions are patterned into individual layers of different thicknesses that can then be assembled into stratified PCMs. This would open up a way to the generation of PCMs with internalized complex communication pathways or to the microscale engineering of soft machines and devices that comprise localized components to carry out specific biosynthetic tasks.

The communication properties of the PCMs were then investigated by assembling PCMs capable of an internalized GOx/HRP enzyme cascade. These PCMs were capable of sensing the external environment and triggering a coordinated internalized biochemical response using an endogenously produced signaling molecule (H_2_O_2_). The unique communication properties of these materials were then employed to construct for the first time arrays of synthetic tissues that were capable of dynamically extracting encoded information provided by the external environment in the form of unidirectional diffusing fronts of chemical species.

Our results open up a route from the synthetic construction of different protocell building blocks with adhesion capabilities to their programmed assembly and spatial integration into cm‐sized tissue‐like materials with precise architectures and geometries. These PCMs are stable in water and are capable of combining the specialization of individual protocell types with the emergent spatiotemporal biochemical response of the ensemble.

From a more general perspective, the programmed assembly of non‐equilibrium materials capable of emerging bioinspired functions from protocell building blocks addresses important challenges of bottom‐up synthetic biology and biomimetic materials science and is expected to open new avenues toward novel organized platforms for tissue engineering, personalized therapy, pharmacokinetics, micro‐bioreactor technologies, and soft robotics. For example, we envision the possibility of engineering PCMs capable of integrating with living cells and tissues, interacting both through chemical and mechanical stimuli to influence cell growth, proliferation and differentiation or to provide targeted therapies. Our floating mold technique could be used to program the assembly of specific protocell phenotypes into arrays of biomimetic organoids that could be used to study the spatiotemporal diffusion and distribution of drugs. PCM arrays could also be chemically programmed to perform continuous biocatalytic synthetic tasks and deliver (bio)molecules of interest. We also envision the possibility of utilizing the techniques outlined in this work to engineer soft robots (e.g., swimmers and walkers) and soft robotic components (e.g., sensors and valves) from specialized protocells capable of chemo‐mechanical transduction.

To conclude, the possibilities that our paradigm shifting approach to prototissue engineering opens up are many. Most importantly, the PCMs described here provide a highly modular platform to both start tackling important fundamental scientific challenges (e.g., understanding of the physicochemical basis of collective and emergent behaviors of living tissues) and to facilitate the development of new protocell applications through their spatial integration into tissue‐like materials endowed with higher‐order coordinated functions.

## 4 Experimental Section

Detailed description of the materials and instruments used, experimental procedures and methods are provided in the Supporting Information. This includes the preparation of fluorescently labeled bio‐orthogonal proteinosomes, assembly of PCMs, PCM patterning and stratification, generation of enzymatically active PCMs, and the investigation of the PCMs’ dynamical sensing of propagating concentration gradients.

## Conflict of Interest

The authors declare no conflict of interest.

## Supporting information

Supporting Information

Supplemental Video 1

Supplemental Video 2

Supplemental Video 3

Supplemental Video 4

Supplemental Video 5

Supplemental Video 6

Supplemental Video 7

Supplemental Video 8

Supplemental Video 9

## Data Availability

The data that support the findings of this study are available from the corresponding author upon reasonable request.
