# Supplementary material for: A Floating Mold Technique for the Programmed Assembly of Protocells into Protocellular Materials Capable of Non‐Equilibrium Biochemical Sensing
Source: Adv Mater. 2021 May 7;33(24):2100340. doi: 10.1002/adma.202100340 (PMC11469123; doi:10.1002/adma.202100340)
Supplement: Supplementary file 1 — Supporting Information [file ADMA-33-2100340-s008.pdf]

© 2021 Wiley-VCH GmbH

ADVANCED MATERIALS

Supporting Information

for *Adv. Mater.,* DOI: 10.1002/adma.202100340

A Floating Mold Technique for the Programmed Assembly of Protocells into Protocellular Materials Capable of Non-Equilibrium Biochemical Sensing

*Agostino Galanti, Rafael O. Moreno-Tortolero, Raihan Azad, Stephen Cross, Sean Davis, and Pierangelo Gobbo**

Supporting Information

A floating mold technique for the programmed assembly of protocells into protocellular materials capable of non-equilibrium biochemical sensing

Agostino Galanti,[1] Rafael O. Moreno-Tortolero,[1] Raihan Azad,[1] Stephen Cross,[2] Sean Davis,[1] and Pierangelo Gobbo[1]*

[1] *School of Chemistry, University of Bristol, Bristol, BS8 1TS (UK).*
E-mail: pierangelo.gobbo@bristol.ac.uk
[2]*Wolfson Bioimaging Facility, Biomedical Sciences Building, University of Bristol, Bristol, BS8 1TD (UK)*

Table of Contents

1. Supplementary methods 2
1.1 Materials and methods 2
1.2 Preparation of fluorescently labelled and non-labelled azide- and BCN-functionalized proteinosomes in oil 5
1.3 Programmed assembly of fluorescently labelled protocellular materials (PCMs) 6
1.4 Kinetics of PCM assembly and transfer to the aqueous phase 7
1.5 Programmed assembly of fluorescently labelled patterned PCMs 7
1.6 Programmed assembly of fluorescently labelled stratified PCMs 8
1.7 Generation of FITC-labelled or non-labelled GOx-containing BCN-functionalized proteinosomes in oil and of non-labelled HRP-containing azide-functionalized proteinosomes in oil 9
1.8 Investigation of the chemical communication properties of enzymatically active PCMs 9
1.9 Dynamical sensing of propagating concentration gradients within arrays of enzymatically active PCMs 10
2. Supplementary figures 13
3. Supplementary videos 22
4. References 24

1. Supplementary methods

1.1 Materials and methods

All reagents were used as received. Glucose oxidase (GOx) (50 KU), horseradish peroxidase (HRP) (25 KU), O,O′-bis[2-(N-succinimidyl-succinylamino)ethyl]polyethylene glycol (PEG-diNHS, M_n = 2,000 Da), 2-ethyl-1-hexanol, *o*-phenylenediamine (*o*-PD), ethanol, and polysorbate 80 were purchased from Sigma- Aldrich. Amplex Red was purchased from Invitrogen (Thermo-Fisher Scientific). α-D-glucose (Glc) was purchased from VWR. Carboxymethyl dextran (CM-dextran, M_n 150,000 Da) was purchased from TdB Labs. Milli-Q water (resistivity at 25 °C = 18.2 MΩ cm) was generated using a Milli-Q Reference Water Purification System with nozzle equipped with an RNA filter.

Dylight405 NHS-ester (Thermo-Fisher Scientific), fluorescein isothiocyanate (FITC, Sigma-Aldrich), or rhodamine isothiocyanate (RITC, Sigma-Aldrich)-labelled and non-labelled azide- and BCN-functionalized BSA/PNIPAM-co-MAA nanoconjugates were synthesized and characterized according to the general procedure established previously.[1] Non-bio-orthogonally reactive FITC- and RITC-labelled BSA/PNIPAM-co-MAA nanoconjugates were synthesized using the same procedure, with the exception that the final step of bio-orthogonal group conjugation was not carried out.

PTFE molds were laser-cut from PTFE sheets 0.5 mm thick. The following mold geometries were built and used in this work:

- 4x4 circle array: circle diameter = 2 mm; distance between circles = 3 mm.
- Square, side 5 mm.
- Equilateral triangle, side 10 mm.
- “Gobbo Group” logo, font: Arial Black (see Figure 2).
- “Gobbo Group” logo, font: Athelas (see Figure S6, Supporting Information).
- “Gobbo Group” logo, font: Arial Black (see Figure S7, Supporting Information).
- “Gobbo Group” logo, font: Courier New (see Figure S8, Supporting Information).

Low resolution fluorescence microscopy was performed using a Dinolite AM4115T-GFBW USB microscope for green fluorescence (480 nm LED for excitation, 510 nm emission filter). Multiple images were acquired by manually moving the samples on the microscope stage. The images were stitched together using ImageJ software to create a larger field of view.

Widefield fluorescence microscopy images were obtained using a Leica DMI 6000 inverted epifluorescence microscope with a Photometrics Prime 95B sCMOS camera and a Leica DFC365FX monochrome camera. The instrument was equipped with a motorized stage that enabled multi-position image acquisition and Adaptive Focus Control (AFC) system ensuring that the specimen was actively kept in focus during a time course. Excitation was provided by a metal halide lamp with fluorescence excitation and emission selected by use of appropriate filter cubes as follows: Dylight405 λ_{exc} = 360/40 nm, dichroic mirror = 400 nm, λ_{em} = 470/40 nm; FITC λ_{exc} = 470/40 nm, dichroic mirror = 495 nm, λ_{em} = 525/50 nm; RITC λ_{exc} = 545/26 nm, dichroic mirror = 565 nm, λ_{em} = 605/70 nm. Images were acquired with a Leica HCX PL Fluotar 5x/0.15NA, HC PL Fluotar 10x/0.32NA, or HCX PL Fluotar 20x/0.4NA lens with multiple images per sample obtained and then stitched together creating a larger field of view. The image stitching process was performed automatically with LASX software (Leica).

Confocal fluorescence microscopy images were obtained using a Leica SP5-II laser scanning microscope attached to a Leica DMI 6000 inverted epifluorescence microscope, with a Leica DFC7000 T-lamp, equipped with the following lasers: 50 mW 405 nm diode laser, 150 mW Ar laser (458, 476, 488, 514 nm lines), 20 mW solid state yellow laser (561nm), and 20 mW Red He/Ne (633 nm) laser. Images were obtained using a 10x/0.4NA lens at a zoom level of 2.6 with either 1024 x 1024 pixels or 512 x 512 pixels of resolution. When z-stacks were performed, images were acquired with a z-step between slices of 10 µm. Excitation was provided by either a 405 nm laser (Dylight405), 488nm Argon laser (FITC) or 561 nm laser (RITC). The following emission wavelengths (λ_{em}) were monitored for the corresponding probes: Dylight405 λ_{em} = 420 nm, FITC λ_{em} = 525 nm, RITC λ_{em} = 566 nm. Adaptive focus control was used to correct focus drift during time course.

All widefield and confocal fluorescence microscopy measurements were carried out in an environmental chamber maintained at 25°C. To assess any changes between samples, the instrument settings (laser power, exposure time, detector gain etc.) were maintained constant across all experiments. All images were processed and analyzed using ImageJ software.[2,3]

To prepare the samples for SEM analysis, PCMs were placed in water, frozen for 24 hrs at -63°C. Then, samples were freeze-dried in vacuum for at least 24 hrs, leaving behind the dried material. The samples were then placed onto an aluminum stub with a conductive carbon tab attached and the samples were coated with 15 nm thick layer of high purity silver (High Resolution Sputter Coater, Agar Scientific, UK). The SEM micrographs were taken on a JSM-IT300 (JEOL, Japan), operated at 15kV, at a working distance of 26-10 mm, using a secondary electron detector.

Kinetics of PCM transfer to the aqueous were performed using a Canon 70D digital camera equipped with a 50 mm macro lens. Images were acquired from the top every 60 sec for 3 hrs. The resulting time-lapse was then analyzed using MATLAB. All 16 PTFE molds were detected and tracked over time and space using a circular Hough transform and the Munkres algorithm. The mean intensity of all pixels within each well was acquired for the generation of the PCM transfer curves. MATLAB code available at: https://github.com/sjcross/SpotIntensity.

Chemical communication within enzymatically active PCMs was analyzed by widefield or confocal fluorescence microscopy, by monitoring the fluorescence emission of resorufin (FITC λ_{exc} = 470/40 nm or 488 nm, λ_{em} = 525/50 nm; Resorufin λ_{exc} = 545/26 nm or 561 nm, λ_{em} = 605/70 nm). Images were acquired every 66.6 sec for 1 hr. The fluorescence intensity time profiles were integrated using ImageJ software.

Dynamic sensing of chemical gradients propagating through arrays of enzymatically active PCMs was analyzed using a Dinolite AM4115T-GFBW USB microscope for green fluorescence (480 nm LED for excitation, 510 nm emission filter) by monitoring the fluorescence emission of 2,3-diaminophenazine (2,3-DAP). Images were acquired every 60 sec for 8 hrs. The green fluorescence intensity in the videos was converted to a false color scale and the background fluorescence recorded

in the areas surrounding the PCMs was set to zero using ImageJ software. The time-dependent fluorescence intensity response of the PCM array was measured using ImageJ software. The data points were corrected by manual baseline and smoothing using Origin data analysis and graphing software.

In these experiments, we noticed that the excitation light source of the Dinolite AM4115T-GFBW USB fluorescence microscope irradiated the field of view with a slightly higher intensity on the left-hand side. This was due to the geometry of the blue LEDs utilized as the excitation light source. Because of this we were careful in keeping the geometry of the setup the same across all the different dynamic sensing of chemical gradients experiments. This allowed us to compare the relative time-dependent changes in fluorescence intensity across the PCM arrays under the different diffusion/equilibrium conditions.

1.2 Preparation of fluorescently labelled and non-labelled azide- and BCN-functionalized proteinosomes in oil

In a 1.75 ml vial, 30 μL of an aqueous solution of fluorescently labelled or non-labelled azide-functionalized BSA/PNIPAM-co-MAA or BCN-functionalized BSA/PNIPAM-co-MAA nanoconjugates (8 mg mL-1), 10 μL of an aqueous solution of CM-dextran (0.6 M), and 30 μL of a solution PEG-diNHS (67 mg mL-1) in Na2CO3 buffer (pH 8.5, 100 mᴍ) were mixed together. Subsequently, 1 mL of 2-ethyl-1-hexanol was gently added to the aqueous phase at an aqueous/oil volume fraction (φ_w) of 0.07. The mixture was shaken manually for 30 sec to produce a white turbid solution. The Pickering emulsion was readily transferred into an Eppendorf tube where it was left to sediment for at least 3 hrs. Finally, the upper clear oil layer was discarded to leave a concentrated sediment of bio-orthogonally reactive proteinosomes in oil, which were left to crosslink for at least 48 hrs.

1.3 Programmed assembly of fluorescently labelled protocellular materials (PCMs)

In a 30 mm or 47 mm polystyrene Petri dish, 3 mL or 5 mL of an aqueous solution of polysorbate 80 (5 wt%) were added respectively, a PTFE mold was gently placed at the water-air interface and allowed to float. Subsequently, appropriate volumes of concentrated azide- and BCN-functionalized proteinosomes in oil were well mixed in a 1:1 ratio in an Eppendorf tube. In general, a volume of the 1:1 binary proteinosome mixture was then drop-casted into the floating PTFE mold to obtain a volume of 0.64 µL mm^{-2}. The proteinosomes were allowed to transfer into the aqueous solution and self-assemble into a PCM according to the transfer times reported in Table S1, Supporting Information. The edges of the PCM remained attached to the PTFE mold.

For the PCM thickness measurements reported in Figure 1g and Figure S3, Supporting Information, the emulsion volume was systematically varied between 0.08 and 1.10 µL mm^{-2}. The PCM thickness was measured by confocal fluorescence microscopy by scanning on the XZ plane. Multiple images of the thickness of the same PCM were acquired by performing Y-stacks on 8 different XZ planes with a Y-step of 10 µm. The average PCM thickness was calculated as the average of these measurements. For each emulsion volume used, measurements were repeated on 3 freshly prepared samples.

Control experiments to investigate the role of bio-orthogonal chemistry in the PCM programmed assembly process were performed following the same protocol, with the exception that non-bio-orthogonally reactive FITC- and RITC-labelled BSA/PNIPAM-co-MAA nanoconjugates were used instead of fluorescently labelled azide- or BCN-functionalized BSA/PNIPAM-co-MAA nanoconjugates.

Table S1. Table summarizing the experimental parameters employed for the programmed assembly of PCMs using different PTFE moulds.

Type of PTFE mould	Side or diameter (mm)	Area (mm^2)	Emulsion volume (µL)	Approx. transfer time (hrs)
Circle	2	3.1	2.0	3
Square	5	25.0	16.0	6
Equilateral triangle	10	43.3	27.7	8

1.4 Kinetics of PCM assembly and transfer to the aqueous phase

In this experiment a polystyrene Petri dish containing an aqueous solution of polysorbate 80 (5 wt%, 3 mL) and a floating PTFE sheet with a 4x4 array of circular molds (2mm in diameter) was placed on a black sheet of paper which served as the background, see Scheme S1, Supporting Information. Subsequently, increasing volumes of a 1:1 binary proteinosome mixture were drop-casted inside the PTFE circular molds according to Scheme S1, Supporting Information.

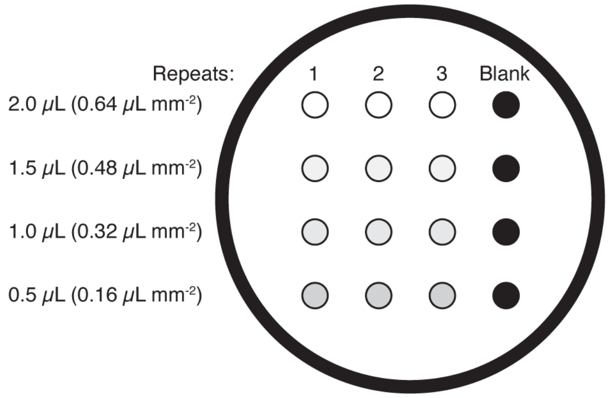

Scheme S1: Scheme showing the 4x4 array of 2 mm circular PTFE molds and describing the different samples prepared.

Kinetics of PCM transfer into aqueous solution were monitored using a Canon 70D digital camera equipped with a macro lens exploiting the visible color change from the white of the emulsion to the black of the background paper, due to the PCMs becoming transparent when completely transferred to the aqueous. The acquired time-lapse was then analyzed using MATLAB to obtain PCM transfer curves and transfer times for the 4 experiments repeated in triplicate (Figure S4 and Video S2, Supporting Information).

1.5 Programmed assembly of fluorescently labelled patterned PCMs

Patterned PCMs with circular and concentric spots were generated using a three-step process following the general procedure described in Section S1.3, Supporting Information. First, a 1:1 mixture of non-labelled azide- and Dylight 405-labelled BCN-functionalized proteinosomes in oil was drop-casted into a square-shaped PTFE mold (5 mm side). Subsequently, a 2 μL droplet of a 1:1 mixture of non-labelled azide- and FITC-labelled BCN-functionalized proteinosomes in oil was drop-

casted in the center of the square. In a third step, a 0.1 μL droplet of a 1:1 mixture of non-labelled azide- and RITC-labelled BCN-functionalized proteinosomes in oil was drop-casted in the center of the first circular drop. The emulsions were manually drop-casted using mechanical pipettes to form the desired patterns. The patterned fluorescently labelled PCMs were finally left to assemble and transfer into the aqueous solution.

Patterned PCMs with 2x2 and 3x3 arrays of protocell consortia were generated using a two-step process following the general procedure described in Section S1.3, Supporting Information. First, a 1:1 mixture of FITC-labelled azide- and FITC-labelled BCN-functionalized proteinosomes in oil was drop-casted into a square-shaped PTFE mold (5 mm side for the 2x2 array and 10 mm side for the 3x3 array). Subsequently, several 0.1 μL droplets of a 1:1 mixture of RITC-labelled azide- and RITC-labelled BCN-functionalized proteinosomes in oil were drop-casted on the background emulsion in order to compose either a 2x2 or a 3x3 array. The emulsions were manually drop-casted using mechanical pipettes to form the desired patterns. The patterned fluorescently labelled emulsions were finally left to transfer into the aqueous solution and assemble into patterned PCMs.

1.6 Programmed assembly of fluorescently labelled stratified PCMs

Fluorescently labelled stratified PCMs were built using a layer-by-layer technique. A first protocell layer was assembled in a circular PTFE mold 2 mm in diameter from a 1:1 mixture of Dylight405-labelled azide- and BCN-functionalized proteinosomes in oil and transferred to water media following the general procedure reported in Section S1.3, Supporting Information. Subsequently, 2 μL of a 1:1 mixture of FITC-labelled azide- and BCN-functionalized proteinosomes in oil were drop-casted on top of the first Dylight405-labelled PCM in water, and the proteinosomes were allowed to transfer to the water media and adhere to the PCM underneath. Finally, 2 μL of a 1:1 mixture of RITC-labelled azide- and BCN-functionalized proteinosomes in oil were drop-casted on top of both the interlinked Dylight405- and FITC-labelled PCM bilayer, and the proteinosomes were allowed to transfer into water media and generate a third PCM layer.

1.7 Generation of FITC-labelled or non-labelled GOx-containing BCN-functionalized proteinosomes in oil and of non-labelled HRP-containing azide-functionalized proteinosomes in oil

In a 1.75 ml vial, 30 μL of an aqueous solution of FITC-labelled BCN-functionalized BSA/PNIPAM-co-MAA nanoconjugate or non-labelled BCN-functionalized BSA/PNIPAM-co-MAA nanoconjugate or non-labelled azide-functionalized BSA/PNIPAM-co-MAA nanoconjugate (8 mg mL-1), 10 μL of an aqueous solution of GOx or HRP (10 μL, 1 mg mL-1), and 30 μL of a solution of PEG-diNHS (67 mg mL-1) in Na2CO3 buffer (pH 8.5, 100 mм) were mixed together. Subsequently, 1 mL of 2-ethyl-1-hexanol was gently added to the aqueous phase at an aqueous/oil volume fraction (φ_w) of 0.07. The mixture was shaken manually for 30 sec to produce a white, turbid solution. The Pickering emulsion was readily transferred into an Eppendorf tube where it was left to sediment for at least 3 hrs. Finally, the upper clear oil layer was discarded to leave a concentrated sediment of enzyme-containing bio-orthogonally reactive proteinosomes in oil, which were let to crosslink for at least 48 hrs.

1.8 Investigation of the chemical communication properties of enzymatically active PCMs

An enzymatically active circular PCM 2 mm in diameter was prepared using the procedure reported in Section S1.3, Supporting Information, with the exception that a 1:1 binary population of GOx-containing FITC-labelled BCN-functionalized proteinosomes in oil and HRP-containing non-labelled azide-functionalized proteinosomes in oil was used instead of non-enzymatically active proteinosomes in oil. Subsequently, an aqueous solution of glucose (2.990 mL, 20 mм) and a freshly prepared solution of Amplex red (10 μL, 150 mм in DMSO) were mixed in a 30 mm polystyrene Petri dish (final glucose and Amplex red concentrations 20 mм and 0.5 mм, respectively). The Petri dish was placed on the microscope stage, which was contained inside an environmental chamber maintained at 25°C. Finally, the enzymatically active PCM attached to a PTFE mold was transferred

into the Petri dish and allowed to float on the substrate solution. The red fluorescence turn-on due to the enzyme-mediated production of resorufin (λ_{exc} = 545/26 nm or 561 nm, λ_{em} = 605/70 nm) was monitored by acquiring images of the PCM every 66.6 sec for 1 hr.

1.9 Dynamic sensing of propagating concentration gradients within arrays of enzymatically active PCMs

To fabricate a 4x4 array of enzymatically active PCMs, a PTFE sheet with a 4x4 array of circular molds (see Figure 4a) was allowed to float on an aqueous solution of polysorbate 80 (5 wt%, 3 mL) in a 35 mm polystyrene Petri dish. The enzymatically active PCMs were assembled by placing 2 µL of a 1:1 binary population of GOx-containing non-labelled BCN-functionalized proteinosomes and HRP-containing non-labelled azide-functionalized proteinosomes in each of the 2 mm circular molds (positions $x_{1\text{-}4}$, $y_{1\text{-}4}$ in Figure 4a). The PCMs were left to transfer to the water media overnight. The day after, the PTFE sheet with the PCM array was gently removed from the transferring solution, the excess liquid was wiped with tissue paper, and the PCM array was transferred to a polystyrene Petri dish containing a phosphate buffer solution (PBS) (10 mм, pH 6.8). The non-equilibrium biochemical sensing experiments were started upon slow injection of a solution of glucose (Glc) and/or *o*-PD in water (*vide infra* for details) in the injection point (Figure 4a). The spatiotemporal response of the 4x4 array of enzymatically active PCMs was monitored over time by recording fluorescence microscopy images (λ_{exc} = 480 nm; λ_{em} = 510 nm) every 60 s for 8 hrs. All experiments were repeated in triplicate.

Glc and o-PD co-diffusion experiment: 20 µL of a solution of Glc (100 mM) and *o*-PD (50 mM) in PBS (10 mM, pH 6.8) were injected through the injection point into 0.980 mL of a PBS (10 mM, pH 6.8).

Table S2: Initial reaction rates (V_0, measured as fluorescence intensity per minute) and onset time of fluorescence signal (*OT*) for the GOx/HRP-mediated cascade reaction measured across the 4x4 array of enzymatically active PCMs. Data obtained under the experimental conditions described above and calculated from Figure S12, Supporting Information.

	x_1		x_2		x_3		x_4	
	V_0 (a.u. min^{-1})	*OT (min)*	*V_0 (a.u. min^{-1})*	*OT (min)*	*V_0 (a.u. min^{-1})*	*OT (min)*	*V_0 (a.u. min^{-1})*	*OT (min)*
y_1	1.7	11.5	1.14	42.5	0.37	109	0.16	176
y_2	1.28	2.57	0.72	42.2	0.13	105	0	na
y_3	1.68	2.1	1.15	18.3	0.28	94.9	0	na
y_4	0.51	1.96	1.72	29.6	1.04	78.4	0.2	147

Preloaded Glc and o-PD experiment: The PTFE sheet with the 4x4 PCM array placed on 1 mL of a solution of Glc (1.0 mM) and *o*-PD (0.5 mM) in PBS (10 mM, pH 6.8).

Table S3: Initial reaction rates (V_0, measured as fluorescence intensity per minute) and onset time of fluorescence signal (OT) for the GOx/HRP-mediated cascade reaction measured across the 4x4 array of enzymatically active PCMs. Data obtained under the experimental conditions described above and calculated from Figure S13, Supporting Information.

	x_1		x_2		x_3		x_4	
	V_0 (a.u. min^{-1})	*OT (min)*	*V_0 (a.u. min^{-1})*	*OT (min)*	*V_0 (a.u. min^{-1})*	*OT (min)*	*V_0 (a.u. min^{-1})*	*OT (min)*
y_1	3.14	2.31	2.49	3.18	2.18	1.25	1.17	0.102
y_2	2.24	1.93	1.18	4.07	1.07	3.13	1.11	1.9
y_3	2.92	0.38	1.62	2.18	1.55	0.17	1.15	3.38
y_4	3.09	1.16	3.14	0.45	2.72	0.816	2.88	0.403

Preloaded Glc and o-PD diffusion experiment: 10 µL of *o*-PD (50 mᴍ) solution in PBS (10 mᴍ, pH 6.8) were injected through the injection point into 0.990 mL of a solution of Glc (1.0 mᴍ) in PBS (10 mᴍ, pH 6.8).

Table S4: Initial reaction rates (V_0, measured as fluorescence intensity per minute) and onset time of fluorescence signal (OT) for the GOx/HRP-mediated cascade reaction measured across the 4x4 array of enzymatically active PCMs. Data obtained under the experimental conditions described above and calculated from Figure S14, Supporting Information.

	x_1		x_2		x_3		x_4	
	V_0 (a.u. min^{-1})	*OT (min)*	*V_0 (a.u. min^{-1})*	*OT (min)*	*V_0 (a.u. min^{-1})*	*OT (min)*	*V_0 (a.u. min^{-1})*	*OT (min)*
y_1	1.34	18.2	0.48	82	0	na	0	na
y_2	2.46	1.88	0.3	40.9	0	na	0	na
y_3	1.85	0.55	0.36	46.3	0	na	0	na
y_4	1.1	20.6	0.52	90.9	0	na	0	na

Preloaded o-PD and Glc diffusion experiment: 10 µL of Glc (100 mᴍ) solution in PBS (10 mᴍ, pH 6.8) were injected through the injection point into 0.990 mL of a solution of *o*-PD (0.5 mᴍ) in PBS (10 mᴍ, pH 6.8).

Table S5: Initial reaction rates (V_0, measured as fluorescence intensity per minute) and onset time of fluorescence signal (OT) for the GOx/HRP-mediated cascade reaction measured across the 4x4 array of enzymatically active PCMs. Data obtained under the experimental conditions described above and calculated from Figure S15, Supporting Information.

	x_1		x_2		x_3		x_4	
	V_0 (a.u. min^{-1})	*OT (min)*	*V_0 (a.u. min^{-1})*	*OT (min)*	*V_0 (a.u. min^{-1})*	*OT (min)*	*V_0 (a.u. min^{-1})*	*OT (min)*
y_1	4.84	6.63	1.02	30.5	0.38	74	0.32	94.9
y_2	4.49	0.6	2.02	17.2	0.26	106	0.28	122.1
y_3	6.28	0.39	2.08	16.4	0.27	97.3	0.34	101
y_4	5.68	6.14	2.84	26.2	0.59	51.3	0.58	48.1

2. Supplementary figures

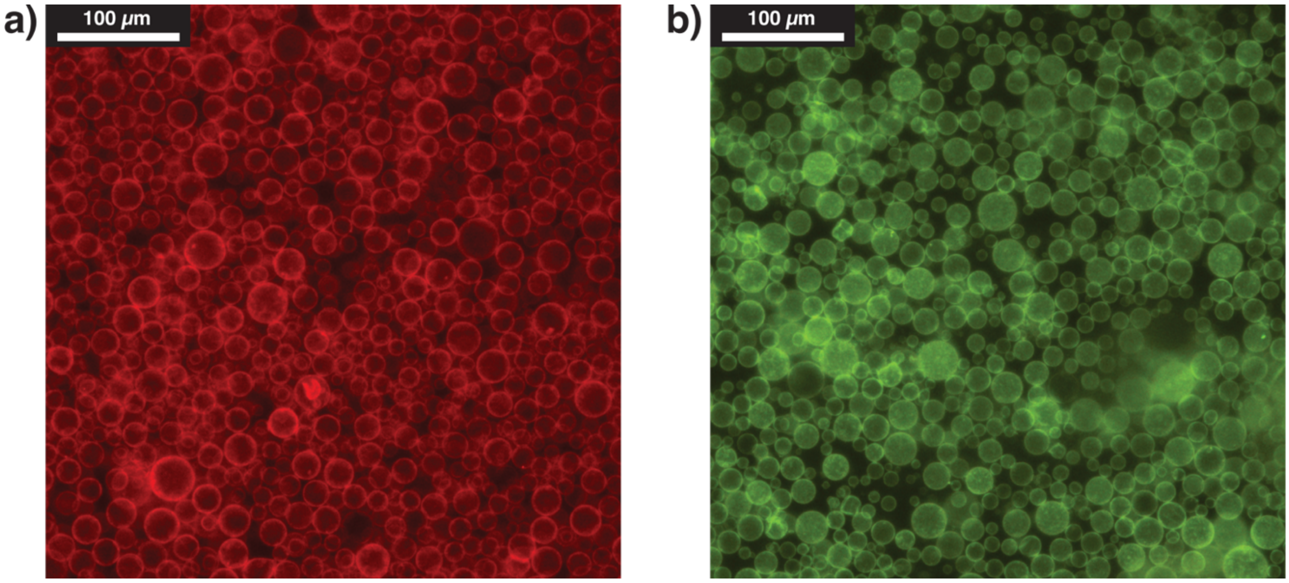

Figure S1: Representative fluorescence microscopy images of bio-orthogonal proteinosomes in oil. a, RITC-labelled azide-functionalized proteinosomes in oil internally crosslinked with PEG-diNHS. Mean diameter: 25 ± 15 µm, 8 ± 2 pL. b, FITC-labelled BCN-functionalized proteinosomes in oil internally crosslinked with PEG-diNHS. Mean diameter: 25 ± 15 µm, 8 ± 2 pL.

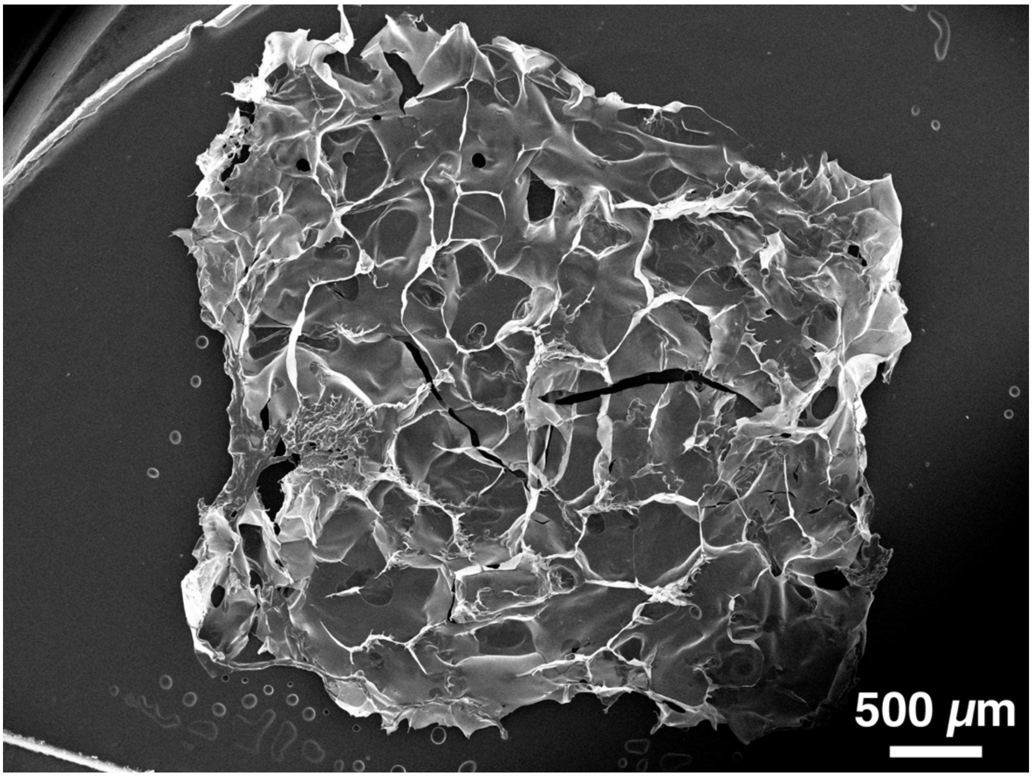

Figure S2: Scanning electron microscopy (SEM) image of a freeze-dried PCM sample. The image highlights the free-standing nature of the PCM.

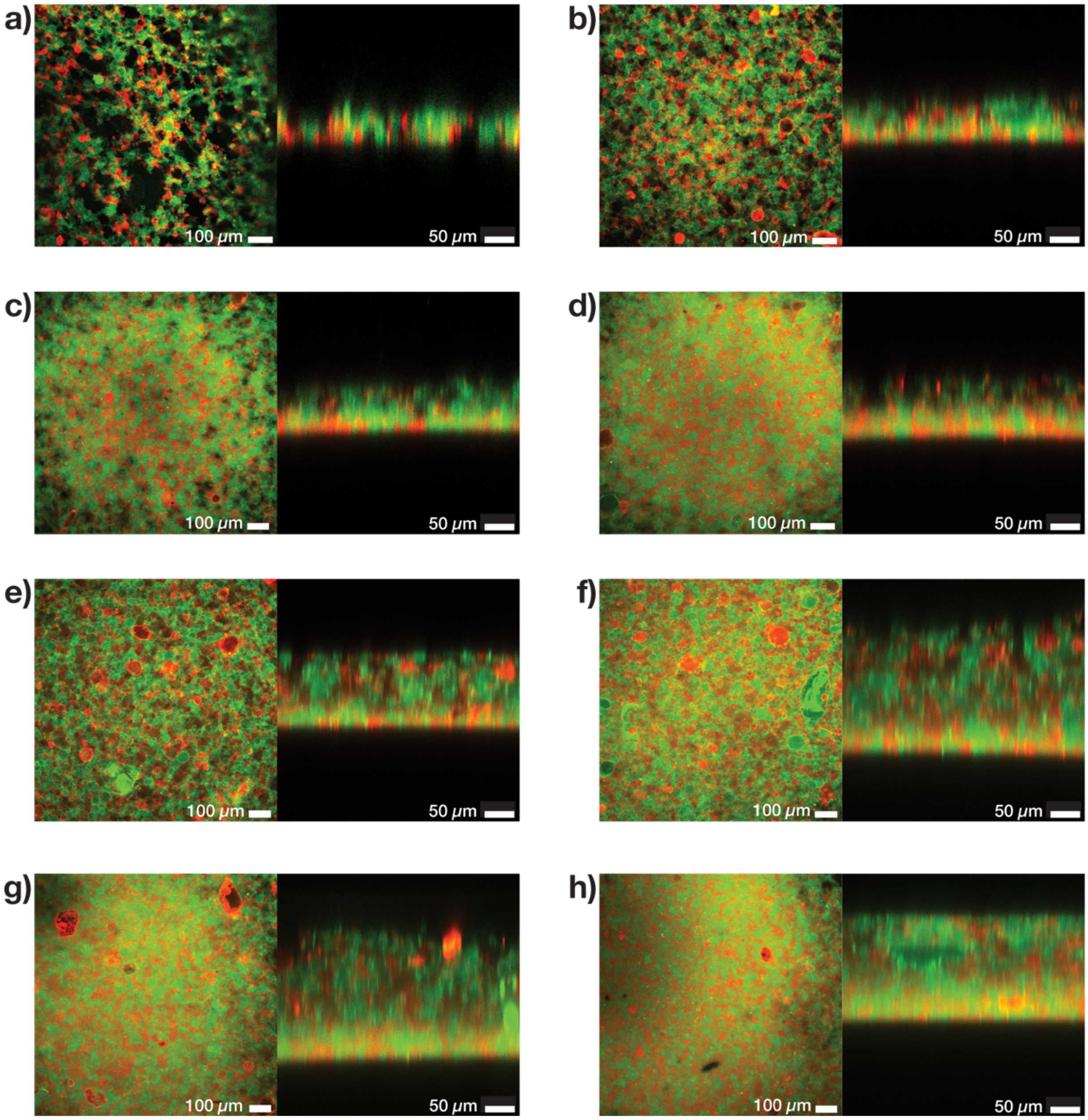

Figure S3: Representative paired confocal fluorescence microscopy images acquired on the XY (left) and XZ plane (right) of PCMs generated using 0.08 μL mm^{-2} (a), 0.16 μL mm^{-2} (b), 0.24 μL mm^{-2} (c), 0.32 μL mm^{-2} (d), 0.48 μL mm^{-2} (e), 0.64 μL mm^{-2} (f), 0.80 μL mm^{-2} (g), 1.10 μL mm^{-2} (h) of emulsion volumes. All emulsions comprised a 1:1 binary population of RITC-labelled (red fluorescence) azide- and FITC-labelled (green fluorescence) BCN-functionalized proteinosomes in oil internally crosslinked with PEG-diNHS. All PCMs were fabricated using a circular mold 2 mm in diameter.

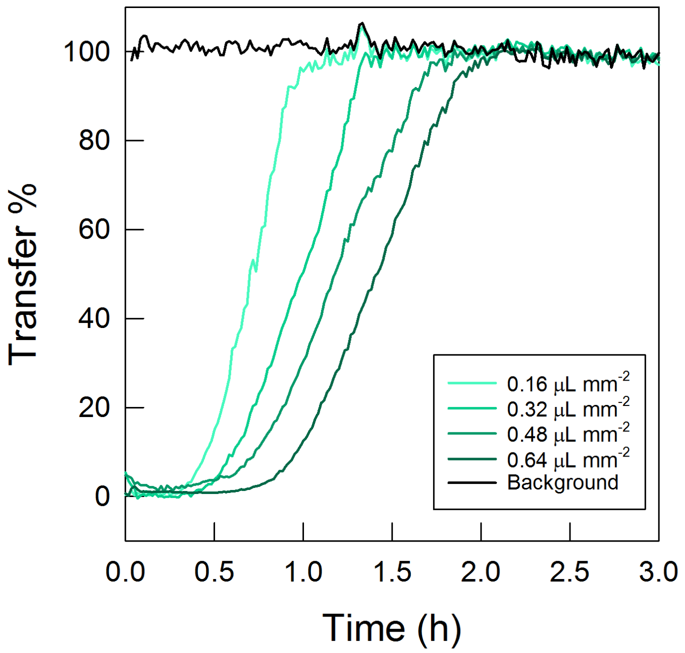

Figure S4: Plot showing oil to water media transfer curves for PCMs generated using a circular PTFE mold 2 mm in diameter and increasing Pickering emulsion volumes of 0.16 to 0.64 µL mm^{-2}. The sigmoidal curves were obtained from the analysis of Video S2, Supporting Information with MATLAB. Each curve is the average of the measurements performed on 3 different PCMs.

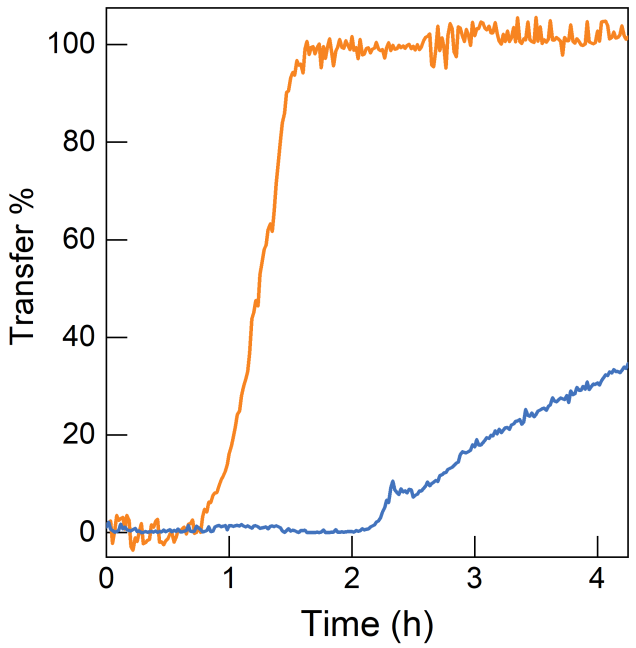

Figure S5: Plot showing oil to water media transfer curves for PCMs generated using a circular PTFE mold 2 mm in diameter and transferred in the presence of a solution 5 wt% of polysorbate 80 in water (orange plot) and in pure water (blue plot).

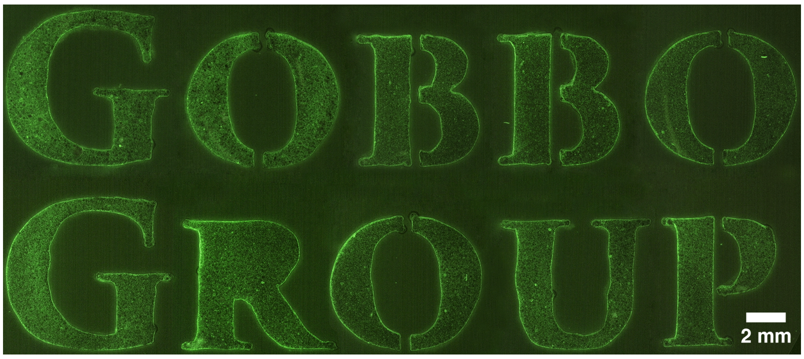

Figure S6: Tiled fluorescence microscopy image (λ_{exc} = 480 nm; λ_{em} = 510 nm) of PCMs with complex shapes obtained using a text-shaped PTFE mold ("Gobbo Group", font: Athelas). The PCMs are composed of an interlinked 1:1 binary population of FITC-labelled BCN-functionalized proteinosomes and RITC-labelled azide-functionalized proteinosomes.

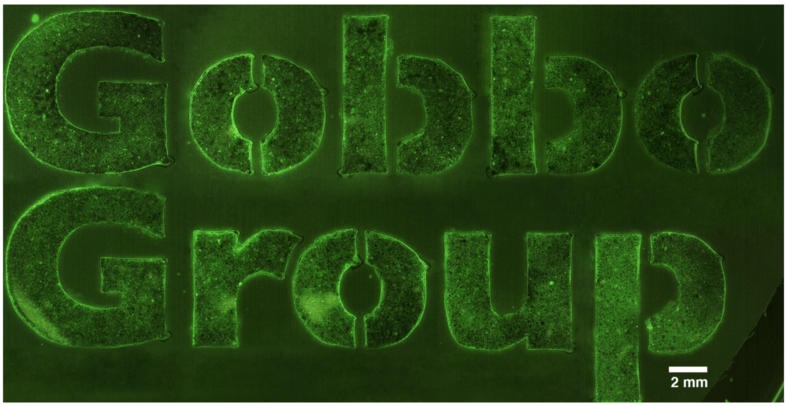

Figure S7: Tiled fluorescence microscopy image (λ_{exc} = 480 nm; λ_{em} = 510 nm) of PCMs with complex shapes obtained using a text-shaped PTFE mold ("Gobbo Group", font: Arial Black). The PCMs is composed of an interlinked 1:1 binary population of FITC-labelled BCN-functionalized proteinosomes and RITC-labelled azide-functionalized proteinosomes.

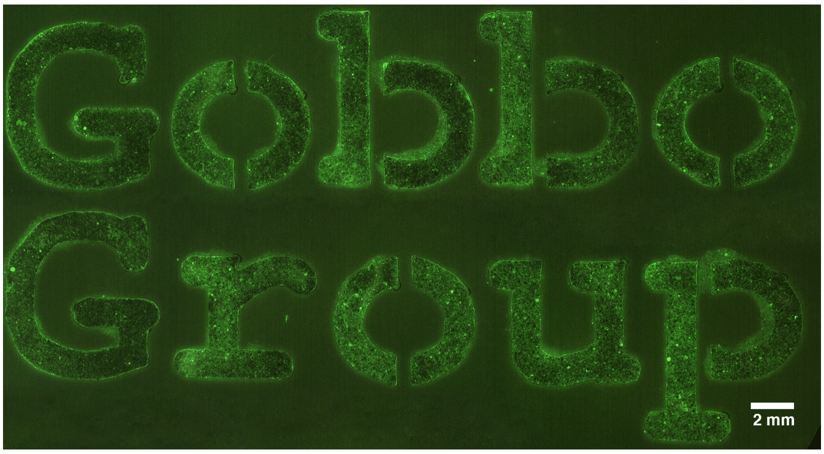

Figure S8: Tiled fluorescence microscopy image (λ_{exc} = 480 nm; λ_{em} = 510 nm) of PCMs with complex shapes obtained using a text-shaped PTFE mold ("Gobbo Group", font: Courier New). The PCMs is composed of an interlinked 1:1 binary population of FITC-labelled BCN-functionalized proteinosomes and RITC-labelled azide-functionalized proteinosomes.

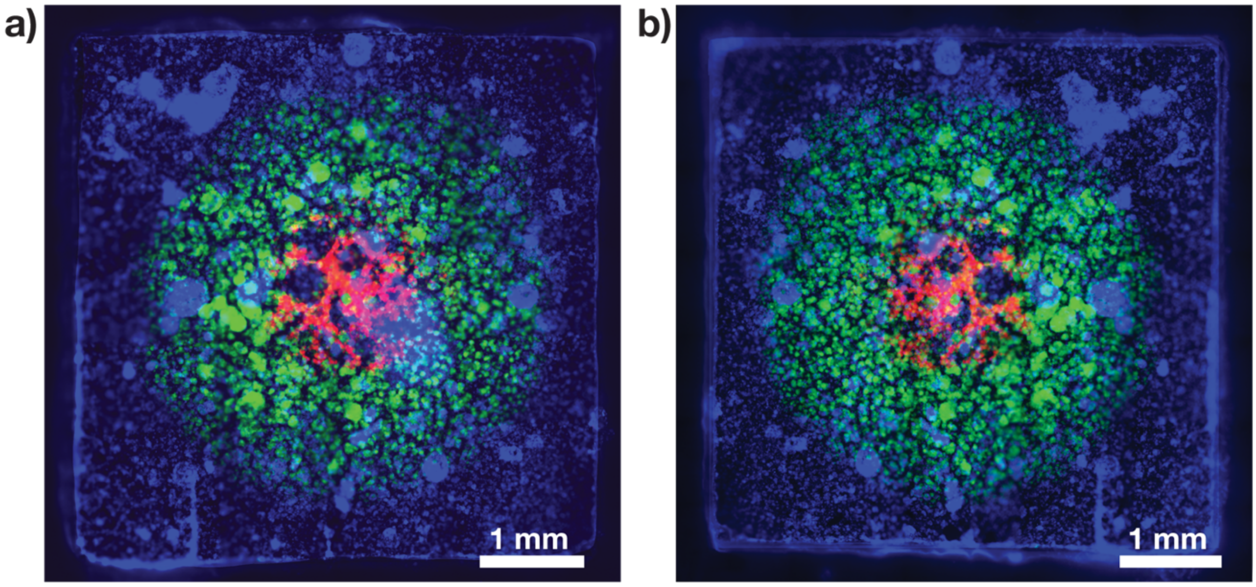

Figure S9: Comparison between the epifluorescence microscopy images of the squared patterned PCM from Figure 2d acquired from the top and from the bottom. a, Tiled epifluorescence microscopy image of the as-prepared patterned PCM. b, Tiled epifluorescence microscopy image acquired after flipping the PCM in (a) upside-down by manipulating it through the PTFE mold. The two images show no substantial differences in the localization of the differently tagged protocell populations. This indicates that our technique produces patterns that are homogenous through the whole PCM thickness, *i.e.,* the pattern remained in the *xy* plane and no stacking of the proteinosome populations was observed.

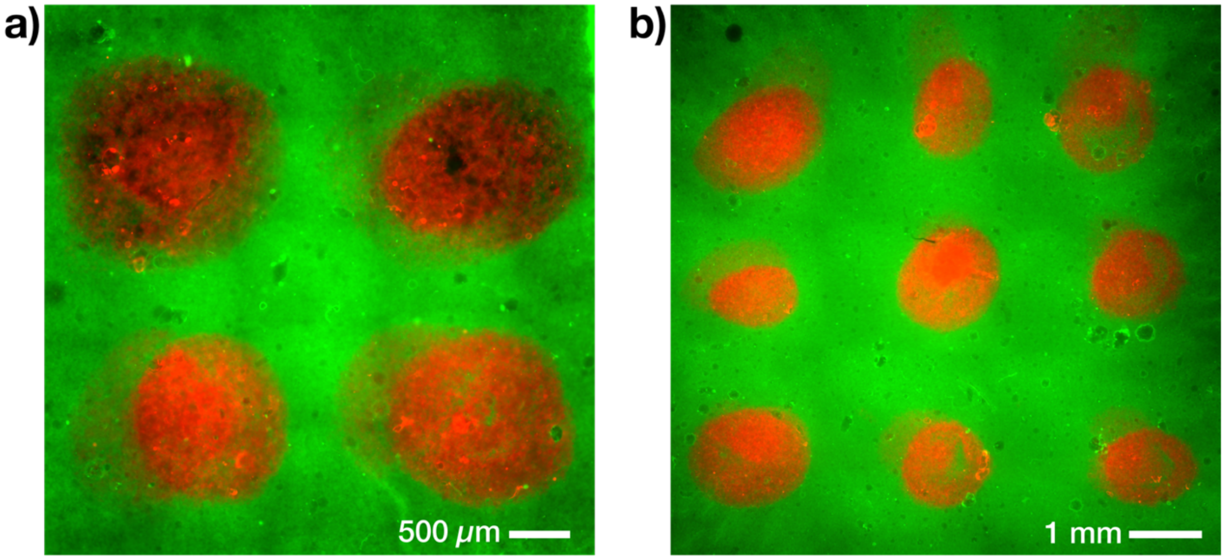

Figure S10: a) Tiled epifluorescence microscopy images of a 5x5 mm PCM patterned with a 2x2 array of bio-orthogonally reactive protocell consortia. b) Tiled epifluorescence microscopy images of a 10x10mm PCM patterned with a 3x3 array of bio-orthogonally reactive protocell consortia. In both figures, green fluorescence: interlinked 1:1 binary populations of FITC-labelled azide- and BCN-functionalized proteinosomes internally crosslinked with PEG-diNHS; red fluorescence: interlinked 1:1 binary populations of RITC-labelled azide- and BCN-functionalized proteinosomes internally crosslinked with PEG-diNHS.

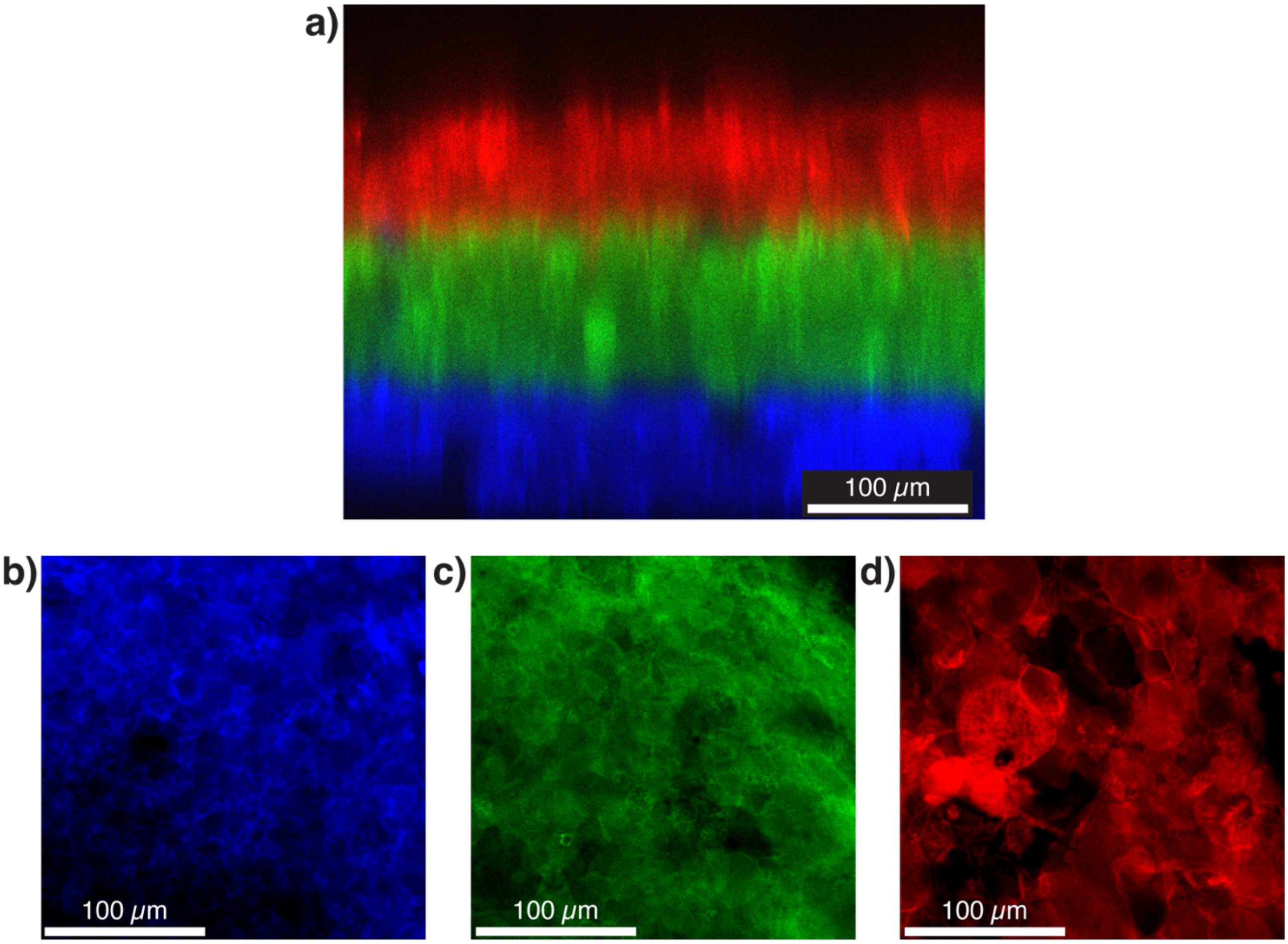

Figure S11: Confocal fluorescence microscopy images of a stratified PCM. a, YZ view of the PCM in Figure 2e showing a prototissue *ca.* 270 µm thick with individual layers *ca.* 90 µm thick. b-d, Images of the individual layers of the stratified PCM in (a). b, Bottom layer of the stratified PCM, consisting of an interlinked 1:1 binary population of Dylight405-labelled bio-orthogonally reactive protocells. c, Middle layer of the stratified PCM, consisting of an interlinked 1:1 binary population of FITC-labelled bio-orthogonally reactive protocells. d, Top layer of the stratified PCM, consisting of an interlinked 1:1 binary population of RITC-labelled bio-orthogonally reactive protocells.

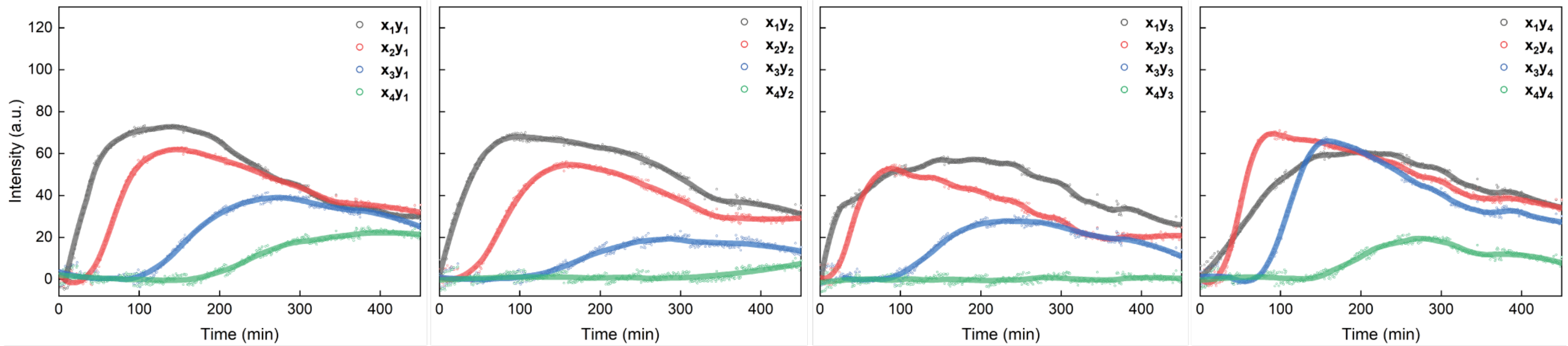

Figure S12: Spatiotemporal responses in a 4x4 array of enzymatically active PCMs obtained by co-diffusing Glc and *o*-PD. Each plot shows the spatiotemporal changes in the 2,3-DAP mean fluorescence intensity (a.u., arbitrary units) for a single row of the 4x4 array of enzymatically active PCMs (from left to right rows y_1 to y_4, see Figure 4a). All 16 PCMs comprised an interlinked 1:1 binary population of non-labelled GOx-containing BCN-functionalized proteinosomes and non-labelled HRP-containing azide-functionalized proteinosomes. The PCMs were attached to the PTFE mold, which was floating on 0.980 mL of PBS (10 mᴍ, pH 6.8) contained in a 35 mm polystyrene Petri dish. The experiment was initiated by injecting 20 µL of a solution of Glc and *o*-PD (100 mᴍ and 50 mᴍ, respectively) into the PBS (below the PCM through the injection point (see Figure 4a). The spatiotemporal changes in the 2,3-DAP mean fluorescence intensity were determined by fluorescence microscopy (λ_{exc} = 480 nm; λ_{em} = 510 nm) by acquiring images every 60 s for 8 hrs. Propagation of the diffusion front of Glc and *o*-PD along the *x* axis resulted in a series of progressively slower initial velocities and increasing activation times for the onset of 2,3-DAP production.

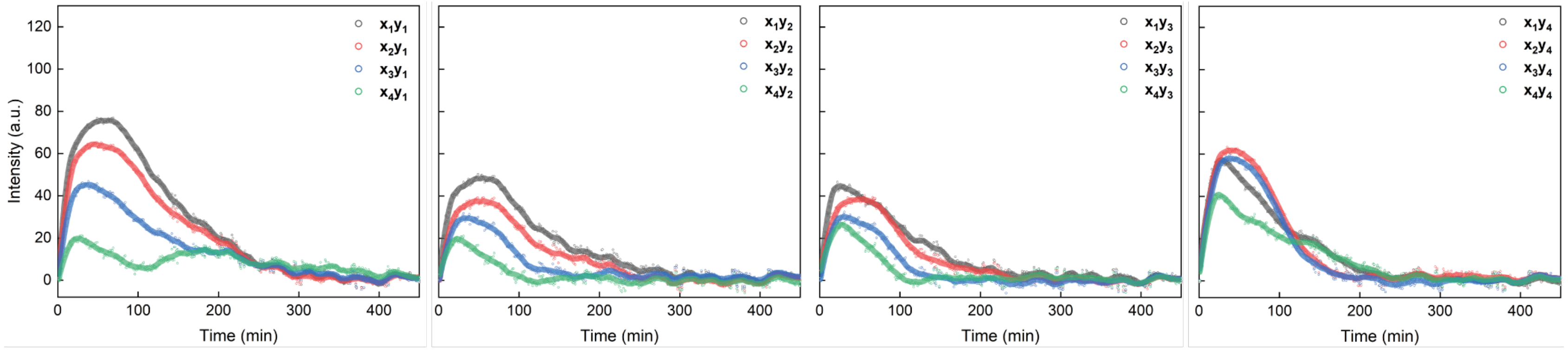

Figure S13: Spatiotemporal responses in a 4x4 array of enzymatically active PCMs obtained under diffusional equilibrium conditions, that is in the absence of a propagating diffusion front of Glc and/or *o*-PD. Each plot shows the spatiotemporal changes in the 2,3-DAP mean fluorescence intensity (a.u., arbitrary units) for a single row of the 4x4 array of enzymatically active PCMs (from left to right rows y_1 to y_4, see Figure 4a). All 16 PCMs comprised an interlinked 1:1 binary population of non-labelled GOx-containing BCN-functionalized proteinosomes and non-labelled HRP-containing azide-functionalized proteinosomes. The PCMs, were attached to the PTFE mold. The experiment was initiated by placing the PTFE mold with attached the 4x4 PCM array on 1 mL of a solution of Glc (1.0 mᴍ) and *o*-PD (0.5 mᴍ) in PBS (10 mᴍ, pH 6.8). The spatiotemporal changes in the 2,3-DAP mean fluorescence intensity were determined by fluorescence microscopy (λ_{exc} = 480 nm; λ_{em} = 510 nm) by acquiring images every 60 s for 8 hrs. Due the absence of a propagating diffusion front, the onset and velocity of 2,3-DAP production was comparable throughout the array of PCMs.

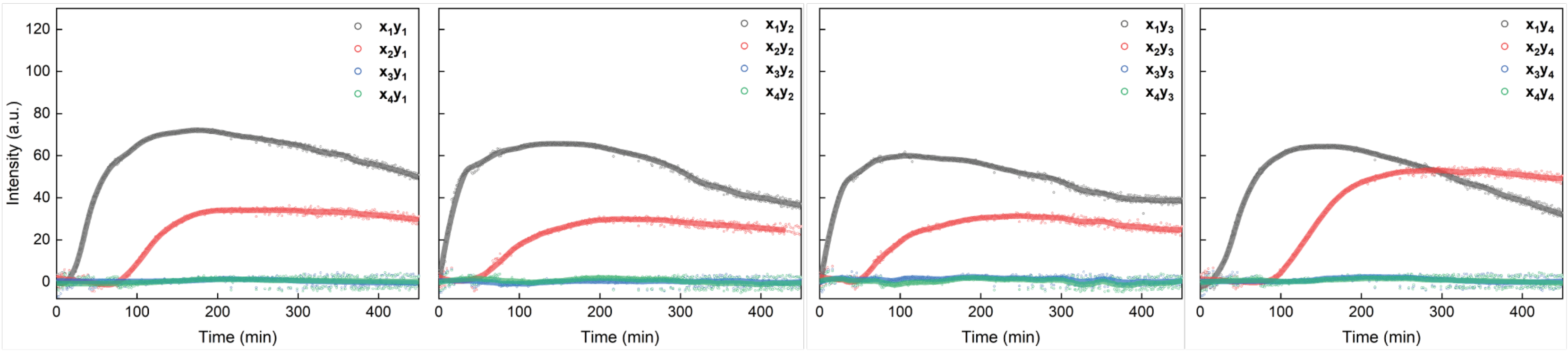

Figure S14: Spatiotemporal responses in a 4x4 array of enzymatically active PCMs obtained by diffusing *o*-PD through a preloaded aqueous solution of Glc. Each plot shows the spatiotemporal changes in the 2,3-DAP mean fluorescence intensity (a.u., arbitrary units) for a single row of the 4x4 array of enzymatically active PCMs (from left to right rows y_1 to y_4, see Figure 4a). All 16 PCMs comprised an interlinked 1:1 binary population of non-labelled GOx-containing BCN-functionalized proteinosomes and non-labelled HRP-containing azide-functionalized proteinosomes. The PCMs were attached to the PTFE mold, which was floating on 0.990 mL of a solution of Glc (1.0 mM) in PBS (10 mM, pH 6.8) contained in a 35 mm polystyrene Petri dish. The experiment was initiated by injecting 10 µL of a solution of *o*-PD (50 mM) through the injection point (see Figure 4a). The spatiotemporal changes in the 2,3-DAP mean fluorescence intensity were determined by fluorescence microscopy (λ_{exc} = 480 nm; λ_{em} = 510 nm) by acquiring images every 60 s for 8 hrs. Propagation of the diffusion front of *o*-PD along the *x* axis resulted in a series of progressively slower initial velocities and increasing activation times for the onset of 2,3-DAP production.

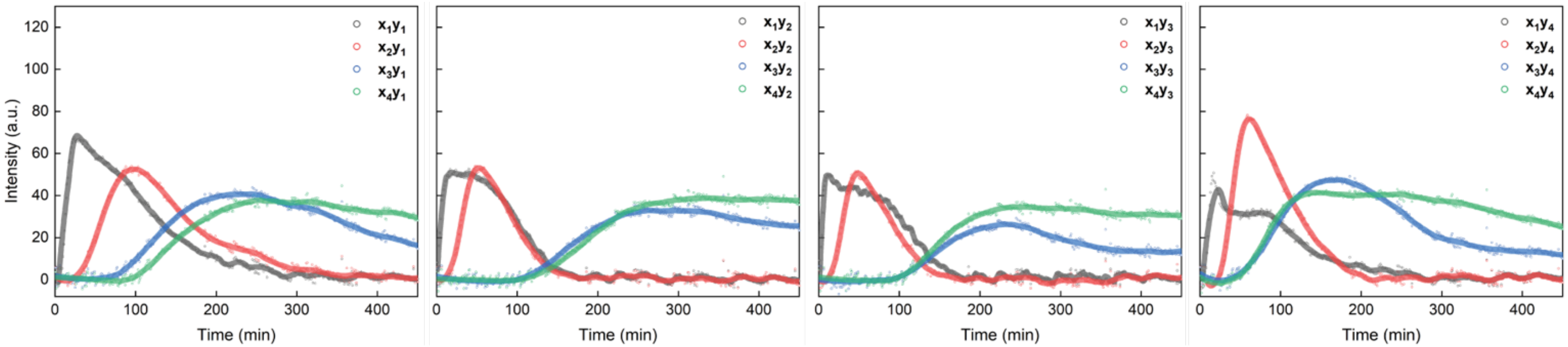

Figure S15: Spatiotemporal responses in a 4x4 array of enzymatically active PCMs obtained by diffusing Glc through a preloaded aqueous solution of *o*-PD. Each plot shows the spatiotemporal changes in the 2,3-DAP mean fluorescence intensity (a.u., arbitrary units) for a single row of the 4x4 array of enzymatically active PCMs (from left to right rows y_1 to y_4, see Figure 4a). All 16 PCMs of the array comprised an interlinked 1:1 binary population of non-labelled GOx-containing BCN-functionalized proteinosomes and non-labelled HRP-containing azide-functionalized proteinosomes. The PCMs were attached to the PTFE mold, which was floating on 0.990 mL of a solution of *o*-PD (0.5 mM) in PBS (10 mM, pH 6.8) contained in a 35 mm polystyrene Petri dish. The experiment was initiated by injecting 10 µL of a solution of Glc (100 mM) through the injection point (see Figure 4a). The spatiotemporal changes in the 2,3-DAP mean fluorescence intensity were determined by fluorescence microscopy (λ_{exc} = 480 nm; λ_{em} = 510 nm) by acquiring images every 60 s for 8 hrs. Propagation of the diffusion front of Glc along the *x* axis resulted in a series of progressively slower initial velocities and increasing activation times for the onset of 2,3-DAP production.

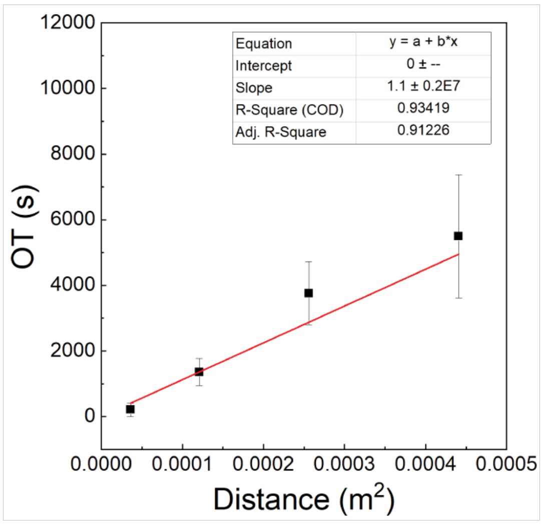

Figure S16: Calculation of the rate of diffusion of glucose. Plot of average fluorescence onset time (OT) for each $x_{1\text{-}4}$ column of the PCM array *vs* distance of the columns from the injection point for the single-component diffusion experiment where Glc was left to diffuse through a preloaded aqueous solution of *o*-PD. The fluorescence OTs were determined by averaging the individual time values determined for the 4 PCMs in each $x_{1\text{-}4}$ column; error bar: standard deviation. The diffusion coefficient of *Glc*, $D = 9.1$ (± 0.2) x 10^{-8} m^2 s^{-1} could be calculated from the reciprocal value of the slope of the linear fitting (red line plot).

3. Supplementary videos

Video S1: Oil removal and PCM assembly process. Fluorescence microscopy video (λ_{exc} = 480 nm; λ_{em} = 510 nm) showing the oil removal process and concomitant PCM programmed assembly in two separate experiments performed by monitoring the Pickering emulsion from above (left) and from the side (right). In both cases the PTFE mold is floating on a solution of polysorbate 80 5 wt% in water and contains a 1:1 binary population of FITC-labelled (green fluorescence) BCN-functionalized and RITC-labelled azide-functionalized proteinosomes in oil with a final volume of 0.64 $\mu L\ mm^{-2}$. The PCM was 2 mm in diameter. To make this video, individual images were acquired every 1 min for 3 hrs and they were mounted in a time-lapse video using ImageJ software.

Video S2: Kinetics of PCM transfer into water media. Animated 3D plot of the spatiotemporal changes in the emulsions' mean intensity value associated with the oil removal and PCM programmed assembly process for the experiment described in Section S1.4, Supporting Information. The appearance of the 3D peaks indicates progressive transfer of the PCMs to the aqueous. The video inset (top right corner) shows on the left the time-lapse video of the experiment, and on the right the real-time tracking of the 16 circular molds 2 mm in diameter (colored circles). The first 3 columns of the 4x4 array from the left contained from top to bottom 0.64, 0.48, 0.32, and 0.16 $\mu L\ mm^{-2}$ of Pickering emulsion comprising 1:1 binary population of crosslinked FITC-labelled BCN-functionalized and RITC-labelled azide-functionalized proteinosomes in oil, respectively. The 4th column on the right was left blank for black background measurements (Scheme S1). The resulting PCM transfer curves of this experiment are reported in Figure S4, Supporting Information.

Video S3: Oil removal and PCM assembly process in the absence of polysorbate 80. Fluorescence microscopy video (λ_{exc} = 480 nm; λ_{em} = 510 nm) showing the oil removal process and concomitant PCM programmed assembly in two separate experiments performed by monitoring the Pickering emulsion from above (left) and from the side (right). In both cases the PTFE mold is floating on MilliQ water and contains a 1:1 binary population of FITC-labelled (green fluorescence) BCN-functionalized and RITC-labelled azide-functionalized proteinosomes in oil with a final volume of 0.64 $\mu L\ mm^{-2}$. In this video growth of large water bubbles on top of the PCM with concomitant PCM deformation and rupture when the bubbles reached critical size can be observed. This still allowed the transfer of the PCMs into water over *ca.* 8 hrs, but the resulting PCMs were more fragile and irregular. To make this video individual images were acquired every 1 min for 8 hrs and were mounted in a time-lapse video using ImageJ software.

Video S4: Oil removal and PCM assembly process using non-bio-orthogonally reactive proteinosomes. Fluorescence microscopy video (λ_{exc} = 480 nm; λ_{em} = 510 nm) showing the oil removal process and concomitant PCM programmed assembly in two experiments carried out in parallel. The experiment on the left was carried out by casting 2 µL of a 1:1 binary population of crosslinked non-bio-orthogonally reactive FITC-labelled and RITC-labelled proteinosomes in oil inside a circular PTFE mold (2 mm in diameter) floating on an aqueous solution of polysorbate 80 (5 wt%). The experiment on the right was instead carried out using 2 µL of a 1:1 binary population of crosslinked FITC-labelled BCN-functionalized and RITC-labelled azide-functionalized proteinosomes in oil. The video on the right shows a PCM transferring mechanism that is similar to that shown in Supplementary Video 1. In contrast, the video on the left clearly shows the Marangoni flow pushing non-reactive proteinosomes incapable of interfacial adhesions towards the edge of the PTFE mold and into the bulk solution. This video clearly shows the critical role of the I-SPAAC reaction in the programmed assembly of PCMs. To make this video, individual images were acquired every 1 min for 3 hrs and were mounted in a time-lapse video using ImageJ software.

Video S5: Investigation of the chemical communication properties of enzymatically active PCMs. Left, Epifluorescence microscopy video (λ_{ex} = 488 nm for FITC; λ_{ex} = 530 nm for resorufin) showing enzyme cascade synthesis of resorufin at 25 °C in a circular PCM (2 mm in diameter). The PCM was assembled from a 1:1 binary population of GOx-containing FITC-labelled BCN-functionalized proteinosomes and HRP-containing non-labelled azide-functionalized proteinosomes. The enzyme cascade reaction was carried out in the presence of a solution of glucose (20 mM) and Amplex red (0.5 mM) in PBS (10 mM, pH = 6.8). Right, Zoomed-in confocal fluorescence microscopy video of the system on the left.
Initially, in both videos only the GOx-containing FITC-labelled BCN-functionalized proteinosome population is visible, followed by development of red fluorescence in the background and within the HRP-containing proteinosomes population. The development of red fluorescence occurs by inter-protocell diffusion of hydrogen peroxide and HRP-mediated reaction with Amplex red to produce resorufin. Diffusion of resorufin from the HRP-containing proteinosomes produces red fluorescence in the neighboring GOx-containing proteinosomes and external aqueous environment. Both videos are shown at 86x real-time speed, the total duration of the recordings was 20 min in real time. Individual frames showing four different stages of the enzyme cascade synthesis of resorufin are reported in Figure 3b.

Video S6: Spatiotemporal responses in a 4x4 array of enzymatically active PCMs obtained by co-diffusing Glc and *o*-PD. Fluorescence microscopy video (λ_{exc} = 480 nm; λ_{em} = 510 nm) showing spatiotemporal changes in the 2,3-DAP mean fluorescence intensity (in false color scale) for a 4x4 array of circular and enzymatically active PCMs 2 mm in diameter. All 16 PCMs comprised an interlinked 1:1 binary population of non-labelled GOx-containing BCN-functionalized proteinosomes and non-labelled HRP-containing azide-functionalized proteinosomes. The PCMs were attached to the PTFE mold, which was floating on 0.980 mL of PBS (10 mM, pH 6.8) contained in a 35 mm polystyrene Petri dish. The experiment was initiated by injecting 20 µL of a solution of Glc and *o*-PD (100 mM and 50 mM, respectively) in the PBS through the injection point on the left of the field of view (see Figure 4a). Co-diffusion of Glc and *o*-PD along the x axis resulted in a progressive turn-on of the 4 PCM columns $x_{1\text{-}4}$ due to a localized GOx-mediated production of hydrogen peroxide, inter-protocell diffusion of hydrogen peroxide and HRP-mediated oxidation of *o*-PD to the green fluorescent molecule 2,3-DAP (Figure S12, Table S2, Supporting Information). The video is shown at 2000x real-time speed; the background signal surrounding the circular PCMs was manually set to zero. Individual frames showing four different stages of the PCM array spatiotemporal response are reported in Figure 4b.

Video S7: Spatiotemporal responses in a 4x4 array of enzymatically active PCMs obtained under equilibrium conditions, that is in the absence of a propagating diffusion front of Glc and/or *o*-PD. Fluorescence microscopy video (λ_{exc} = 480 nm; λ_{em} = 510 nm) showing spatiotemporal changes in the 2,3-DAP mean fluorescence intensity (in false color scale) for a 4x4 array of circular and enzymatically active PCMs 2 mm in diameter. All 16 PCMs comprised an interlinked 1:1 binary population of non-labelled GOx-containing BCN-functionalized proteinosomes and non-labelled HRP-containing azide-functionalized proteinosomes. The PCMs were attached to the PTFE mold. The experiment was initiated by placing the PTFE mold with attached 4x4 PCM array on 1 mL of a solution of Glc (1.0 mM) and *o*-PD (0.5 mM) in PBS (10 mM, pH 6.8). Due the absence of a propagating diffusion front, onset and velocity of 2,3-DAP production was comparable throughout the array of PCMs (Figure S13, Table S3, Supporting Information). The video is shown at 2000x real-time speed; the background signal surrounding the circular PCMs was manually set to zero. Individual frames showing four different stages of the PCM array spatiotemporal response are reported in Figure 4c.

Video S8: Spatiotemporal responses in a 4x4 array of enzymatically active PCMs obtained by diffusing *o*-PD through a preloaded aqueous solution of Glc. Fluorescence microscopy video (λ_{exc} = 480 nm; λ_{em} = 510 nm) showing spatiotemporal changes in the 2,3-DAP mean fluorescence intensity (in false color scale) for a 4x4 array of circular and enzymatically active PCMs 2 mm in diameter. All 16 PCMs comprised an interlinked 1:1 binary population of non-labelled GOx-containing BCN-functionalized proteinosomes and non-labelled HRP-containing azide-functionalized proteinosomes. The PCMs were attached to the PTFE mold, which was floating on 0.990 mL of a solution of Glc (1.0 mM) in PBS (10 mM, pH 6.8) contained in a 35 mm polystyrene Petri dish. The experiment was initiated by injecting 10 µL of a solution of *o*-PD in MilliQ water (50 mM) through the injection point (see Figure 4a). Diffusion of *o*-PD along the *x* axis resulted in a progressive turn-on of the first 2 PCM columns $x_{1,2}$ due to a localized GOx-mediated production of hydrogen peroxide, inter-protocell diffusion of hydrogen peroxide and HRP-mediated oxidation of *o*-PD to the green fluorescent molecule 2,3-DAP (Figure S14, Table S4, Supporting Information). The video is shown at 2000x real-time speed; the background signal surrounding the circular PCMs was manually set to zero.

Video S9: Spatiotemporal responses in a 4x4 array of enzymatically active PCMs obtained by diffusing Glc through a preloaded aqueous solution of *o*-PD. Fluorescence microscopy video (λ_{exc} = 480 nm; λ_{em} = 510 nm) showing spatiotemporal changes in the 2,3-DAP mean fluorescence intensity (in false color scale) for a 4x4 array of circular and enzymatically active PCMs 2 mm in diameter. All 16 PCMs comprised an interlinked 1:1 binary population of non-labelled GOx-containing BCN-functionalized proteinosomes and non-labelled HRP-containing azide-functionalized proteinosomes. The PCMs were attached to the PTFE mold, which was floating on 0.990 mL of a solution of *o*-PD (0.5 mM) in PBS (10 mM, pH 6.8) contained in a 35 mm polystyrene Petri dish. The experiment was initiated by injecting 10 µL of a solution of Glc (100 mM) through the injection point (see Figure 4a). Diffusion of Glc along the *x* axis resulted in a progressive turn-on of the 4 PCM columns $x_{1\text{-}4}$ due to a localized GOx-mediated production of hydrogen peroxide, inter-protocell diffusion of hydrogen peroxide and HRP-mediated oxidation of *o*-PD to the green fluorescent molecule 2,3-DAP (Figure S15, Table S5, Supporting Information). The video is shown at 2000x real-time speed; the background signal surrounding the circular PCMs was manually set to zero.

4. References

[1] P. Gobbo, A. J. Patil, M. Li, R. Harniman, W. H. Briscoe, S. Mann, *Nat. Mater.* **2018**, *17*, 1145.

[2] J. Schindelin, I. Arganda-Carreras, E. Frise, V. Kaynig, M. Longair, T. Pietzsch, S. Preibisch, C. Rueden, S. Saalfeld, B. Schmid, J. Y. Tinevez, D. J. White, V. Hartenstein, K. Eliceiri, P. Tomancak, A. Cardona, *Nat. Methods* **2012**, *9*, 676.

[3] C. T. Rueden, J. Schindelin, M. C. Hiner, B. E. DeZonia, A. E. Walter, E. T. Arena, K. W. Eliceiri, *BMC Bioinformatics* **2017**, *18*, 529.
